# Tertiary Lymphoid Structure-B Cells Narrow Regulatory T Cells Impact in Lung Cancer Patients

**DOI:** 10.3389/fimmu.2021.626776

**Published:** 2021-03-08

**Authors:** Claire Germain, Priyanka Devi-Marulkar, Samantha Knockaert, Jérôme Biton, Hélène Kaplon, Laïla Letaïef, Jérémy Goc, Agathe Seguin-Givelet, Dominique Gossot, Nicolas Girard, Pierre Validire, Marine Lefèvre, Diane Damotte, Marco Alifano, François M. Lemoine, Keith E. Steele, Jean-Luc Teillaud, Scott A. Hammond, Marie-Caroline Dieu-Nosjean

**Affiliations:** ^1^Sorbonne Université, UMRS 1135, Faculté de Médecine Sorbonne Université, Paris, France; ^2^Laboratory “Immune Microenvironment and Immunotherapy”, INSERM U1135, Centre d'Immunologie et des Maladies Infectieuses Paris (CIMI-Paris), Paris, France; ^3^Sorbonne Université, UMRS 1138, Paris, France; ^4^Laboratory “Cancer, Immune Control, and Escape”, INSERM U1138, Cordeliers Research Center, Paris, France; ^5^Université de Paris, UMRS 1138, Paris, France; ^6^Thoracic Department, Curie-Montsouris Thorax Institute, Institut Mutualiste Montsouris, Paris, France; ^7^Université Sorbonne Paris Nord, Sorbonne Paris Cité, Faculté de Médecine SMBH, Bobigny, France; ^8^Oncology Department, Curie-Montsouris Thorax Institute, Institut Curie, Paris, France; ^9^Department of Pathology, Institut Mutualiste Montsouris, Paris, France; ^10^Department of Pathology, Assistance Publique-Hopitaux de Paris (AP-HP), Cochin Hospital, Paris, France; ^11^Department of Thoracic Surgery, Assistance Publique-Hopitaux de Paris (AP-HP), Cochin Hospital, Paris, France; ^12^Oncology Translational Sciences, AstraZeneca, Gaithersburg, MD, United States; ^13^Oncology Research, AstraZeneca, Gaithersburg, MD, United States

**Keywords:** B cell, immune checkpoint, immune microenvironment, lung cancer, regulatory T cell, tertiary lymphoid structure

## Abstract

The presence of tertiary lymphoid structures (TLS) in the tumor microenvironment is associated with better clinical outcome in many cancers. In non-small cell lung cancer (NSCLC), we have previously showed that a high density of B cells within TLS (TLS-B cells) is positively correlated with tumor antigen-specific antibody responses and increased intratumor CD4^+^ T cell clonality. Here, we investigated the relationship between the presence of TLS-B cells and CD4^+^ T cell profile in NSCLC patients. The expression of immune-related genes and proteins on B cells and CD4^+^ T cells was analyzed according to their relationship to TLS-B density in a prospective cohort of 56 NSCLC patients. We observed that tumor-infiltrating T cells showed marked differences according to TLS-B cell presence, with higher percentages of naïve, central-memory, and activated CD4^+^ T cells and lower percentages of both immune checkpoint (ICP)-expressing CD4^+^ T cells and regulatory T cells (Tregs) in the TLS-B^high^ tumors. A retrospective study of 538 untreated NSCLC patients showed that high TLS-B cell density was even able to counterbalance the deleterious impact of high Treg density on patient survival, and that TLS-B^high^ Treg^low^ patients had the best clinical outcomes. Overall, the correlation between the density of TLS-B^high^ tumors with early differentiated, activated and non-regulatory CD4^+^ T cell cells suggest that B cells may play a central role in determining protective T cell responses in NSCLC patients.

## Introduction

The tumor microenvironment is marked by its complexity and substantial amount of heterogeneity. Tumor-infiltrating lymphocytes (TILs) play a role in this heterogeneity and have an important impact on the clinical outcome of cancer patients. Studies have shown that while high densities of TIL CD8^+^ T cells or TIL B cells are associated with better survival in some cancers, other immune subsets, such as regulatory T cells (Tregs), are mostly associated with poor clinical outcomes (1–3). Careful examination of the tumor microenvironment has pointed out the capacity of intratumor immune cells to organize into tertiary lymphoid structures (TLS) (4), ectopic lymphoid structures that appear upon sustained inflammation and are similar in organization to secondary lymphoid organs (SLOs). TLS have a B-cell area composed mainly of follicular B cells (TLS-B cells) and follicular dendritic cells (DCs), adjacent to a T-cell area containing clusters of T cells and mature DCs (TLS-DC) (2, 5, 6). Antigen-specific responses develop in TLS, which are associated with increased severity in autoimmune diseases (7) but have a positive effect during infections by clearing pathogens (8). In tumors, high TLS density is most often associated with better clinical outcomes (4, 9–11). We have previously shown that high densities of TLS-DC or TLS-B cells are associated with prolonged survival in non-small cell lung cancer (NSCLC) (2, 5, 12), and that TLS-B cell^high^ tumors are also linked to the development of tumor antigen-specific antibodies (2) and increased TIL CD4^+^ T cell repertoire clonality (13).

In light of these findings, we used extensive gene expression profiling and flow cytometry for an integrative analysis of the phenotypes of TIL B cells and TIL CD4^+^ T cells in regards of TLS-B density. We show that TIL B cells and TIL CD4^+^ T cells are more highly activated in tumors than in the periphery and that they express key ligand/receptor pairs necessary for B/T interactions and two-way co-stimulation. Moreover, a high density of TLS-B cells is associated with higher frequencies of activated CD4^+^ T cells and lower frequencies of both immune checkpoint (ICP)-expressing CD4^+^ T cells and regulatory T cells (Tregs) in the intratumor CD4^+^ T cell compartment. High densities of TLS-B cells together with low densities of FoxP3^+^ Tregs in NSCLC tumors consistently identified the group of patients with the best clinical outcome.

Overall, these results suggest that TLS-B cells promote the development of protective CD4^+^ T cell-mediated immune responses.

## Materials and Methods

### Patients

This retrospective study examined formalin-fixed, paraffin-embedded (FFPE) primary NSCLC samples from 538 patients who underwent surgery without any neoadjuvant chemoradiotherapy between 2001 and 2005 at Hôtel Dieu Hospital in Paris, France (Supplementary Table 1). We also used 56 fresh tumor biopsies, retrieved prospectively at the Institut Mutualiste Montsouris and Cochin Hospital (Paris, France), to study gene expression (*n* = 26) or perform flow cytometry analyses (*n* = 30, associated when possible with non-tumor lung biopsies and/or peripheral blood analyses) (Supplementary Table 2). Patients who had received neoadjuvant chemoradiotherapy were ineligible. Serial sections of the corresponding FFPE NSCLC tumors were obtained for these 56 patients, together with a written informed consent before their inclusion in the study. The local ethics and human investigation committees (n° 2008-133, 2012-0612, and 2017-A03081-52) approved these protocols, in application of article L.1121-1 of the French Public Health Code. Peripheral blood was obtained from healthy volunteers at the *Etablissement Français du Sang* (EFS, Paris, France, n°15EFS012 and n°18EFS033).

### Immunohistochemistry

FFPE NSCLC blocks were selected, and sections were stained as previously described (2, 12, 14), under the antigen retrieval conditions and with the antibodies and reagents listed in Supplementary Table 3. Slides were digitally scanned with a Nanozoomer (Hamamatsu), operated with NDPview software.

### Method for Cell Quantification

Cells from the entire tumor section were quantified with Calopix® software (Tribvn). Because TLS-B cells were highly aggregated (Supplementary Figures 1A,B), their density was measured and expressed as a percentage of the whole tumor area:

[Total surface of CD20^+^ TLS-B cells (mm^2^)/Total tumor area (mm^2^)]^*^100.

CD3^+^ FoxP3^+^ T cells (Supplementary Figure 1D) were described by their cell density, i.e., the absolute number of cells/mm^2^ of tumor area, as previously reported (12). Both immunostaining and quantification were reviewed by at least two independent observers among CG, JG, SK, PDM, and M-CD-N.

### Flow Cytometry

Mononuclear cells were recovered from fresh lung specimens or blood, and multiple-parameter flow cytometry analysis was performed as previously described (14), with the reagents and antibodies listed in Supplementary Table 4. Cells were acquired on an LSR Fortessa cell analyzer (BD Biosciences) by applying the gating strategy presented in Supplementary Figure 2. Results were analyzed with DIVA (BD Biosciences) and FlowJo software (TreeStar, Inc.) for Boolean analyses. Multiple phenotypes were represented as pie charts, with Pestle and Spice software (Mario Roederer, NIAID).

### Cell Sorting and Gene Expression Analysis

CD3^+^ CD4^+^ CD8^−^ T cells were sorted from lung tumor mononuclear cells on a FACSAria III cell sorter (BD Biosciences) as previously described (13, 14), with the reagents and antibodies listed in Supplementary Table 4. Purity was >98%. Total RNA was extracted with the RNeasy Mini Kit (Qiagen SAS, Courtaboeuf, France). Digital multiplexed gene expression analysis used the NanoString nCounter system (PanCancer Immune Profiling Panel, NanoString Technologies), with 4 ng of total RNA from each sample, after pre-amplification, as previously described (13, 14). Genes with geomean counts before normalization below a threshold determined on background, i.e., <20 geomean counts, were excluded from subsequent analysis. Raw data were normalized with nSolver software (NanoString Technologies), based on the 10 most relevant of 39 housekeeping genes.

### Statistical Analysis

For each cell population of interest, patients were stratified into two groups according to its density—high or low. This group was determined by using either the median density (0.623011805% for CD20^+^ TLS-B cells and 39.211937000 cells/mm^2^ for CD3^+^ FoxP3^+^ Tregs) or the optimal *p*-value approach (0.3256612% for CD20^+^ TLS-B cells, and 21.93277 cells/mm^2^ for CD3^+^ FoxP3^+^ Tregs), as previously described (2, 12) (Supplementary Figure 3). Overall survival (OS) curves were estimated by the Kaplan-Meier method, and differences between patient groups were calculated with the log-rank test, corrected according to the formula proposed by Altman *et al*. (15). Univariate and multivariate analyses used the Cox regression model after testing the proportional hazards assumption (PHA *P*-value). Statistical analyses used StatView and R software. A *p* value < 0.05 was defined as statistically significant.

In flow cytometry experiments, depending on data distribution (Shapiro normality test) and whether the observations were matched or not, the differences between the quantitative variables across the different groups were compared by the Kruskal-Wallis, one-way analysis of variance, Friedman, Mann-Whitney, or Wilcoxon (paired, non-Gaussian) tests, with appropriate adjustments for *post-hoc* comparisons (GraphPad Prism 5 software). A *p*-value < 0.05 was defined as statistically significant.

In the Nanostring analysis, correlations were evaluated by a Spearman test. A Benjamini–Hochberg correction was applied to determine the associated false discovery rates (FDRs). Only FDRs <0.10 were considered statistically significant.

## Results

### A High Density of TLS-B Cells Is Associated With a Specific Intratumor CD4^+^ T Cell Gene Expression Signature

As our previous study showed that the presence of TLS-B cells in the tumor microenvironment mainly favors intratumor CD4^+^ T cell clonal expansion (13), we analyzed the expression of 550 immune-related genes in sorted TIL CD4^+^ T cells from 26 NSCLC patients in relation to their TLS-B cell densities.

TLS-B cell density was correlated with 11 genes expressed by TIL CD4^+^ T cells (Supplementary Figure 4A). Among them, 3 genes were positively correlated with TLS-B cell density: *LY96* (*Lymphocyte antigen 96)*, despite its weak expression (Supplementary Figure 4B); *MERTK*, a tyrosine kinase receptor involved in T cell survival and differentiation following TCR activation; and *POU2AF1 (POU class 2 associating factor 1*), a transcriptional co-activator also induced after TCR triggering and contributing to germinal center (GC) formation, to IFN-γ and IL-2 promoter activities (16), and required for robust CD4^+^ T cell memory responses. Conversely, eight genes were negatively correlated with TLS-B cell density (Supplementary Figure 4A). Four of them were highly expressed by CD4^+^ T cells (Supplementary Figure 4B): *PIK3CG* and *MAP2K1*, both involved in cell growth, survival, and proliferation; *ITGB1*, related to cell adhesion; and *CD5*, which plays a role in Treg differentiation (17). The other four genes encoded the adhesion molecule *CD58*; the chemokine *XCL2*; the transcription factor *POU2F2*; and *DPP4*, a receptor involved in TCR-mediated T cell co-activation and Treg-mediated immunosuppression.

Taken together, these results demonstrate that TLS-B cell density correlates with a specific transcriptional gene signature associated with Th1/memory response and GC formation, as well as with decreased proliferative capacities, chemotaxis, and Treg functions in tumor-infiltrating CD4^+^ T cells.

### Specific Phenotypic Profile of Tumor-Infiltrating B Cells Compared With B Cells at Non-tumor Sites

**Figure 1:**
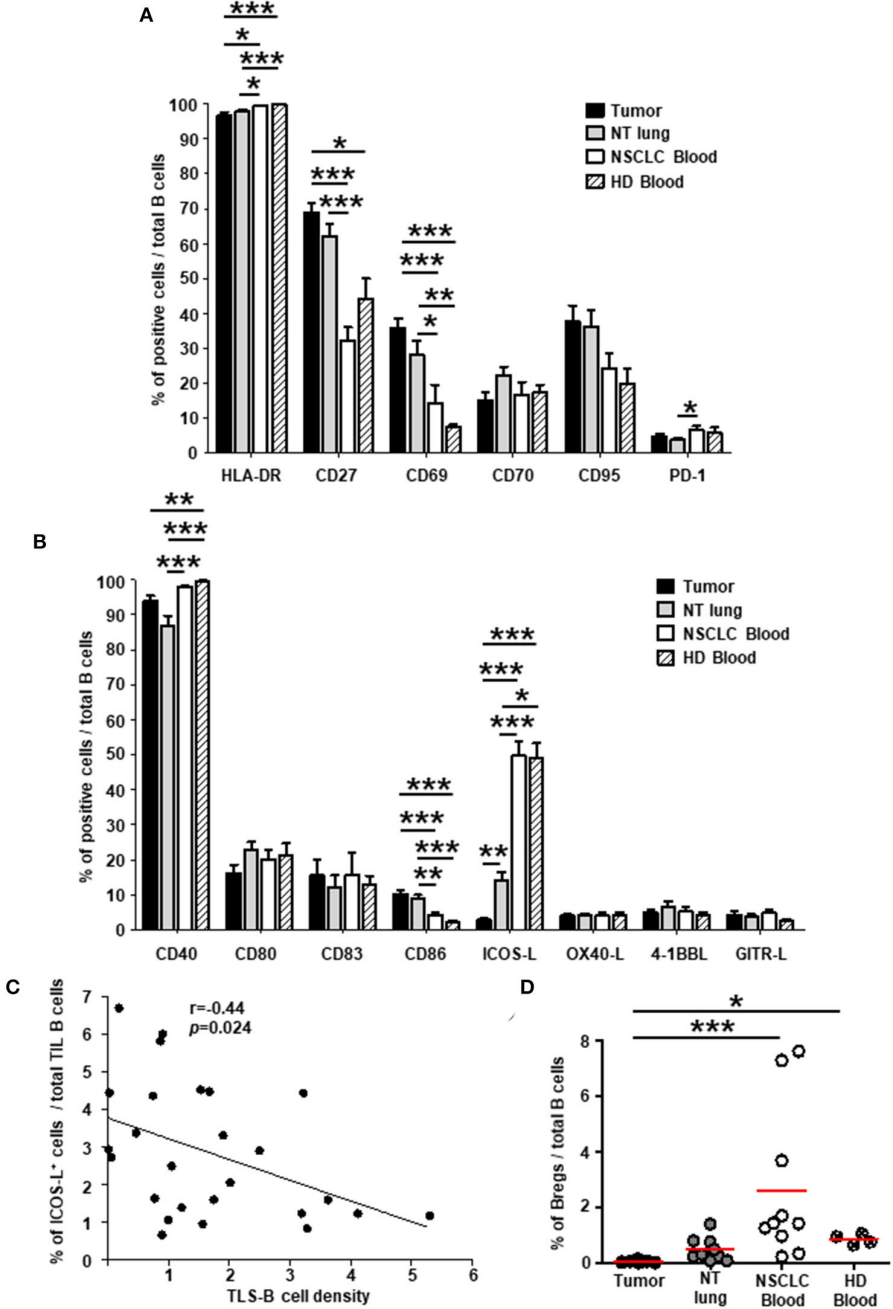
Higher percentages of activated B cells and lower percentages of ICOS-L^+^ B cells in NSCLC tumors compared with distant non-tumor sites. Histograms represent the frequencies of B cells expressing **(A)** activation markers and **(B)** co-stimulatory molecules studied among total B cells in NSCLC tumors, non-tumor (NT) lung, NSCLC peripheral blood, and healthy donor (HD) peripheral blood (mean ± SEM) sites. **(C)** Correlation between the percentage of ICOS-L^+^ B cells among total TIL B cells and TLS-B cell density in the corresponding tumors (*n* = 26). **(D)** Graph represents the percentages of regulatory B cells (Bregs) among total B cells within each compartment. Means are indicated by horizontal red lines. **(A,B,D)** *p*-values were calculated with one-way ANOVA/Kruskal-Wallis/Dunn's test. **(C)** Statistical test used: Spearman test. *p < 0.05, **p < 0.005, ***p < 0.001. HD, healthy donor; NSCLC, non-small-cell lung cancer; NT, non-tumor; TIL, tumor-infiltrating lymphocyte.

We then analyzed the expression of cell surface molecules by TIL B cells in lung tumors compared with those at distant sites, i.e., non-tumor (NT) lung and blood samples from NSCLC patients, and blood from healthy individuals. Most B cells expressed MHC-class II (Figure 1A) and CD40 (Figure 1B) molecules in all the tissues we studied. Consistent with the greater frequency of memory B cells and plasma cells in tumors than in blood (Supplementary Figure 5), a higher percentage of CD27^+^ B cells was also detected in tumors than in blood (Figure 1A and Supplementary Figure 6A, upper panels). The percentages of B cells expressing CD69 (Figure 1A) (mostly activated IgD^−^ memory B cells, Supplementary Figure 6A, middle panels), and CD86 (Figure 1B) (mainly transitional B cells and GC B cells, Supplementary Figure 6B) were significantly higher in tumors than in blood. Of note, the expression of CD80 and CD83 remained similar at the different sites (range = 10-20%). By contrast, the percentage of ICOS-L^+^ B cells were significantly lower in tumors compared with NT sites (Figure 1B) and was negatively associated with TLS-B cell density (Figure 1C). Consistently and as in NSCLC lymph nodes, very low percentages of ICOS-L^+^ naïve and transitional pre-GC IgD^+^ B cells were observed in tumors compared with NSCLC patient blood samples (Supplementary Figure 6A, lower panels). Finally, regulatory B cells, defined as CD38^high^ CD24^high^, were barely detected in tumors (<0.1% of total B cells), compared with NT sites (Figure 1D and Supplementary Figure 6C).

### Expression of Activation, Co-stimulatory Receptors, and ICPs by TIL CD4^+^ T Cells

We then analyzed and compared the expression of activation, co-stimulatory and immunosuppressive molecules by CD4^+^ T cells in NSCLC vs. NT sites.

**Figure 2:**
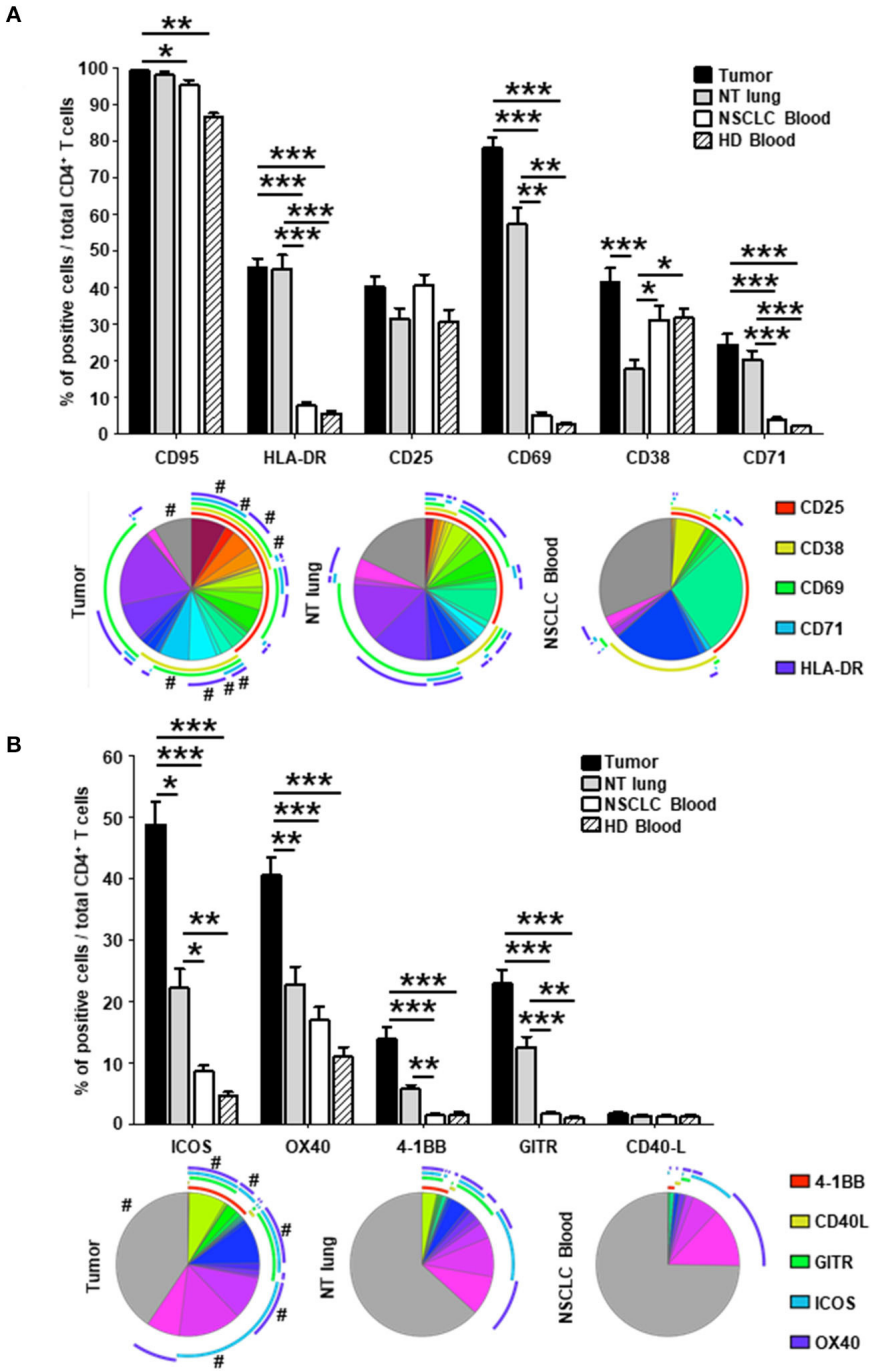
Higher percentages of activated CD4^+^ in tumors vs. NT sites. Histograms represent the percentages of CD4^+^ T cells expressing activation markers **(A)** and co-stimulatory molecules **(B)** among total CD4^+^ T cells in NSCLC tumor, non-tumor (NT) lung, NSCLC peripheral blood and healthy donor (HD) peripheral blood (mean ± SEM) sites. *P*-values were calculated with one-way ANOVA/Kruskal-Wallis/Dunn's test. *p < 0.05, **p < 0.005, ***p < 0.001. The pie charts below each histogram depict the proportion of CD4^+^ T cells with each distinct marker profile in each compartment. The highly expressed CD95 marker (>85% of CD95^+^ CD4^+^ T cells in every compartment) was not included in the analysis. The arcs around each pie chart represent the individual markers studied, as noted in the arc legend. The pie slice colors represent the different combinations of markers observed. A # at the periphery of the pie slices indicates a statistically significant difference (*p* < 0.05) for that individual marker combination between NSCLC tumor, NT lung and NSCLC peripheral blood, determined by a Wilcoxon sign rank test. HD, healthy donor; NSCLC, non-small-cell lung cancer; NT, non-tumor.

As previously observed (12), a greater frequency of effector-memory (EM) CD4^+^ T cells was observed in tumors than in NSCLC NT sites or in blood from healthy donors (HD) (Supplementary Figure 7). The percentage of CD4^+^ T cells not expressing activation markers was lower in tumors compared with NT lung and NSCLC blood (gray sections of the pie charts, Figures 2A,B). Higher percentages of cells expressing HLA-DR, CD69, CD38, and CD71 were detected in tumors than in NT lung and blood from healthy donors and NSCLC patients (Figure 2A, upper panel). Of intratumor CD4^+^ T cells, 78% expressed CD69, and half were triple positive CD69^+^ CD38^+^ HLA-DR^+^. Although the percentage of CD25^+^ cells was similar between the different sites, most CD25^+^ T cell subsets in tumors, contrary to those in blood, co-expressed several activation markers (Figure 2A, lower panel).

**Figure 3:**
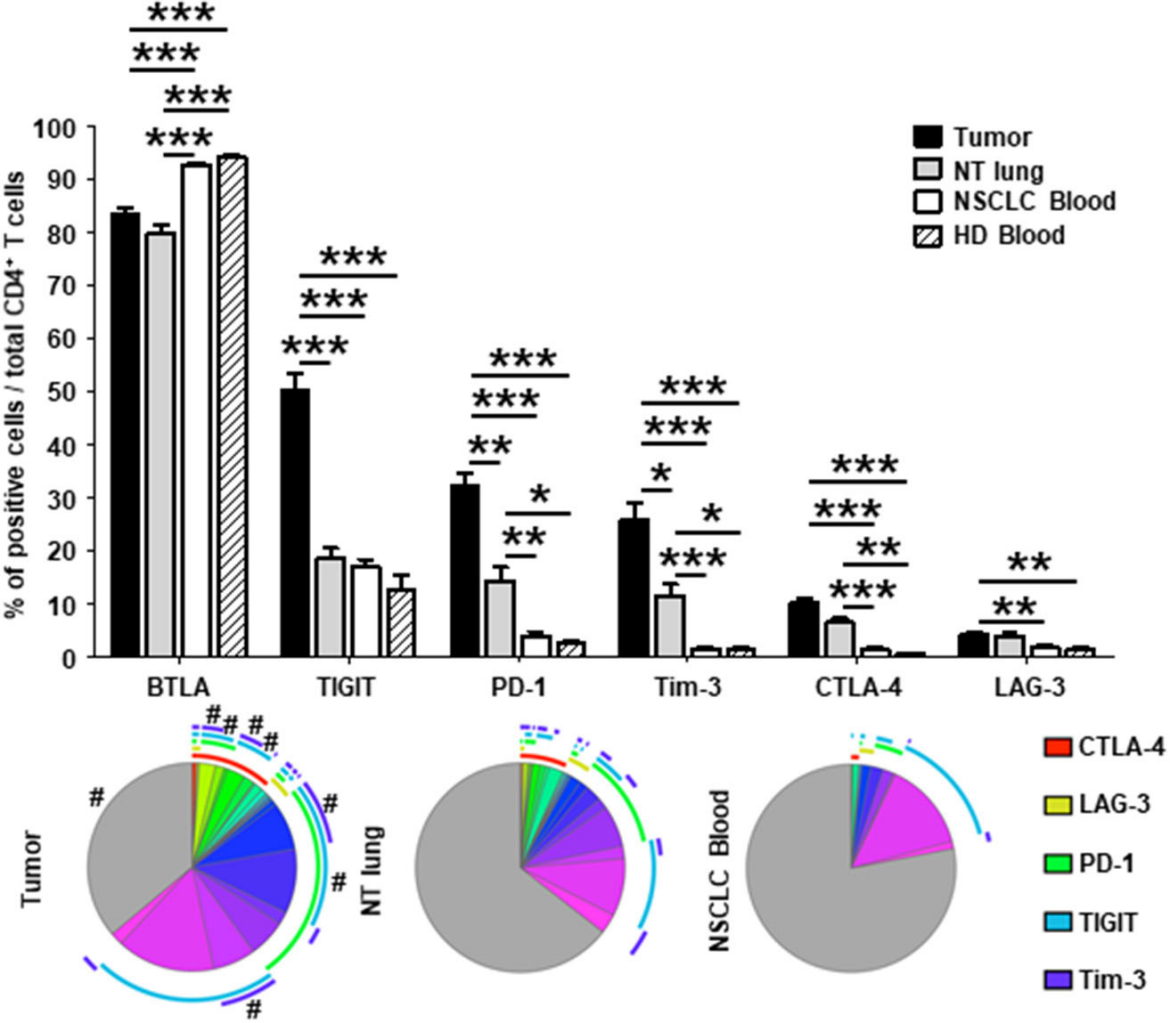
Higher percentages of CD4^+^ T cells expressing immune checkpoints in tumors *vs*. NT sites. Histograms represent the percentages of CD4^+^ T cells expressing immune checkpoints among total CD4^+^ T cells in NSCLC tumor, non-tumor (NT) lung, NSCLC peripheral blood and healthy donor (HD) peripheral blood (mean ± SEM). *P* values were calculated with one-way ANOVA/Kruskal-Wallis/Dunn's test. *p < 0.05, **p < 0.005, ***p < 0.001. The pie charts below each histogram depict the proportion of CD4^+^ T cells with each distinct marker profile in each compartment. The arcs around each pie represent the individual markers studied, as noted in the arc legend. The highly expressed BTLA marker (>80% of BTLA^+^ CD4^+^ T cells in every compartment) was not included in the analysis. The pie slice colors represent the different combinations of markers observed. A # at the periphery of the pie slices indicates a statistically significant difference (*p* < 0.05) for that individual marker combination between NSCLC tumor, NT lung, and NSCLC peripheral blood sites, as determined by a Wilcoxon sign rank test. HD, healthy donor; NSCLC, non-small-cell lung cancer; NT, non-tumor.

The percentage of CD4^+^ T cells that did not express co-stimulatory receptors was significantly lower in tumors than in either NT lung or NSCLC blood (44 vs. 68% and 74%, respectively; Figure 2B, lower panel). In particular, CD4^+^ T cells were positive for ICOS, OX40, 4-1BB, and GITR more frequently in tumors than in the other two sites. Interestingly, only 35% of CD4^+^ T cells in tumors did not express ICP, compared with those from NT lung (almost 65%) and blood (more than 75%) (Figure 3, lower panel). The percentages of cells expressing at least one ICP were higher in tumors than at NT sites (except for BTLA).

Taken together, these results demonstrate that CD4^+^ T cells expressing molecules involved in B-T cell interactions (i.e., co-activation and inhibitory receptors) are present in higher percentages in tumors than at NT sites.

### A High Density of TLS-B Cells Is Associated With Lower Frequencies of Immune Checkpoint-Expressing CD4^+^ T Cells in Tumors

**Figure 4:**
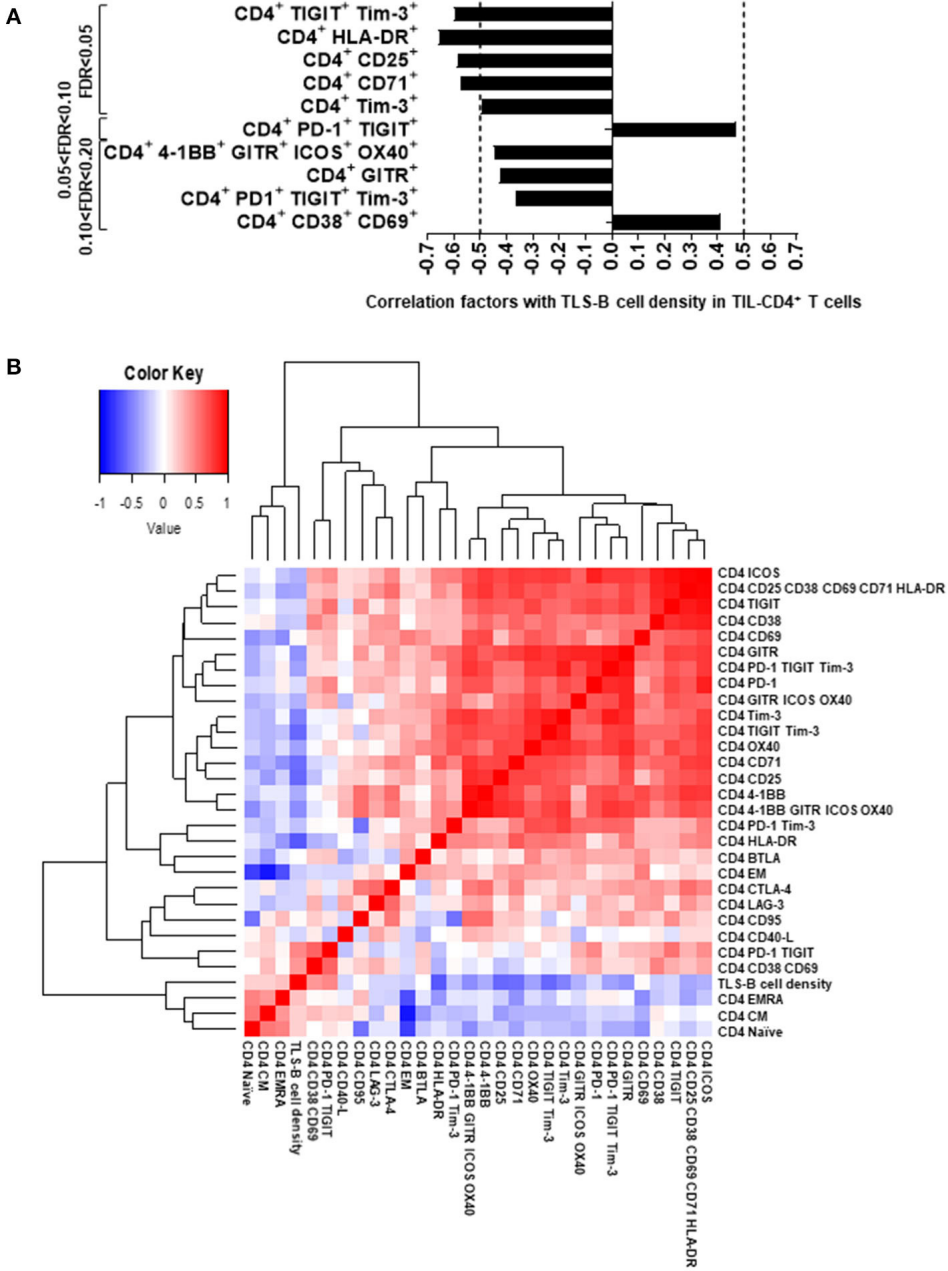
Correlations between TLS-B cell density and marker expression on TIL CD4^+^ T cells. **(A)** Correlations between marker expression on TIL CD4^+^ T cells and TLS-B cell density. Markers significantly correlated with TLS-B cell density (FDR < 0.05, 0.05 < FDR < 0.10, and up to 0.10 < FDR < 0.20) are shown. Vertical dashed lines represent limits for correlation factors (< -0.5 and >+0.5). Statistical test used: Spearman test. **(B)** Heat-map of the markers evaluated by flow cytometry on TIL CD4^+^ T cells together with TLS-B cell density. Statistical test used: Spearman test. Only markers expressed significantly differently at NSCLC tumor, distant NT lung and NSCLC peripheral blood (Figures 2, 3) were included in the analyses. FDR, false discovery rate; NSCLC, non-small-cell lung cancer; NT, non-tumor; TIL, tumor-infiltrating lymphocyte; TLS, tertiary lymphoid structure.

We next examined the relationship between the presence of TLS-B cells (assessed by IHC) and the phenotype of fresh intratumor CD4^+^ T cells (analyzed by flow cytometry) on the corresponding tumor biopsy. TLS-B cell density correlated positively with the percentages of activated [CD38^+^ CD69^+^] CD4^+^ T cells and [PD-1^+^ TIGIT^+^] CD4^+^ T cells (Figure 4A and Supplementary Figure 8), and correlated negatively with the percentages of CD4^+^ T cells that were HLA-DR^+^, CD25^+^, CD71^+^, Tim-3^+^ CD4^+^ T cells, and exhausted [TIGIT^+^ Tim-3^+^], and, to a lesser extent, with the percentages of GITR^+^ and [4-1BB^+^ GITR^+^ ICOS^+^ OX40^+^] CD4^+^ T cells (Figure 4A and Supplementary Figure 8).

Remarkably, TLS-B cell density clustered with naïve, CM, and EMRA CD4^+^ T cells (Figure 4B and Supplementary Figure 7A); this finding suggests that an active T cell homing, differentiation, and activation program may take place in TLS-B^high^ tumors. Conversely, most ICP and Treg markers, including CD25, GITR, CTLA-4, Tim-3, and TIGIT, were associated with clusters distinct and distant from the TLS-B cell cluster (Figure 4B).

These results, taken together, emphasize that high TLS-B cell density in the lung tumor-infiltrating CD4^+^ T cell compartment correlates positively with naïve, CM, and activated cells and negatively with immunosuppressed T cells and Tregs.

### A High Density of TLS-B Cells Is Associated With Lower Treg Frequency in the NSCLC Tumor-Infiltrating CD4^+^ T Cell Compartment

**Figure 5:**
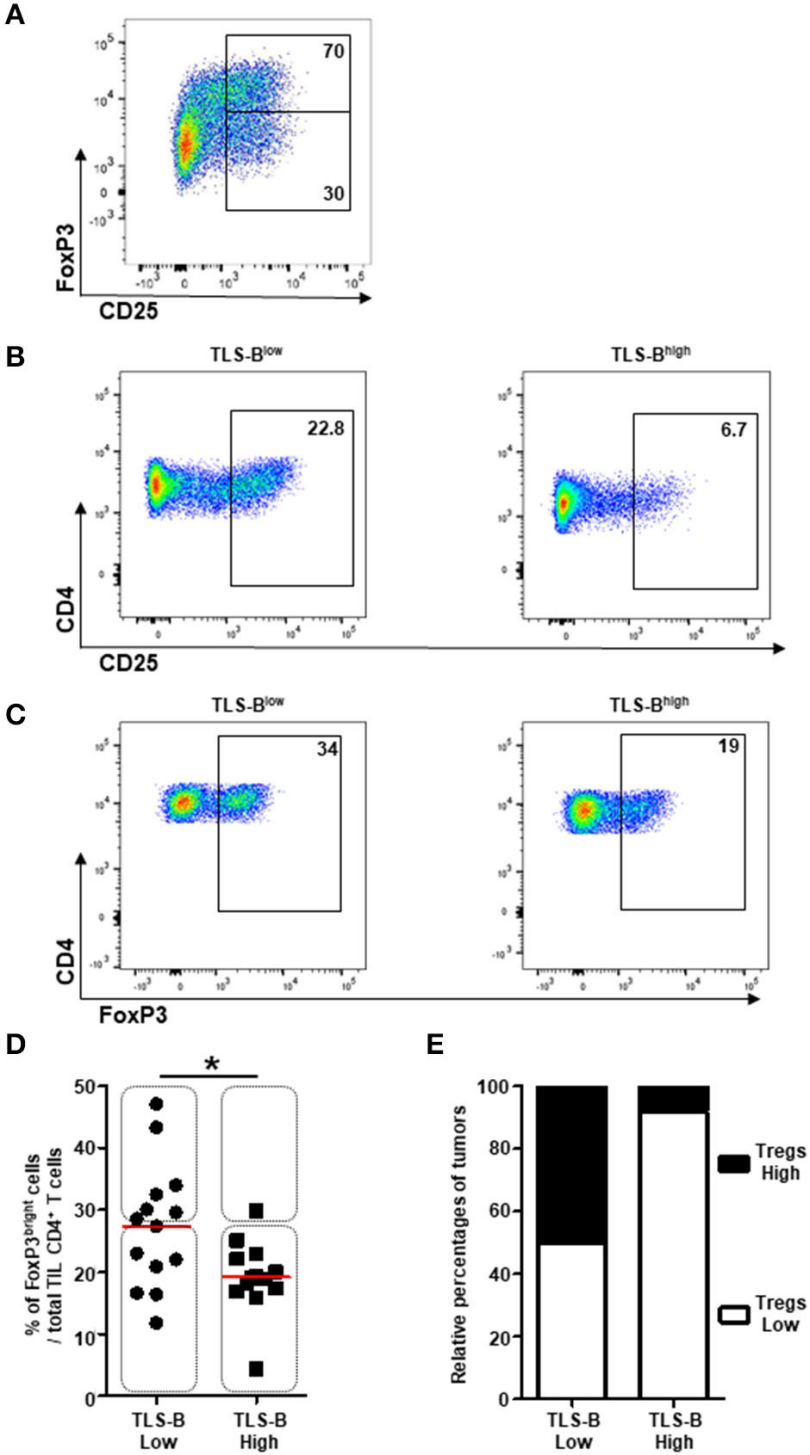
Decreased frequencies of CD4^+^ FoxP3^+^ Tregs in TLS-B^high^ tumors. **(A)** Representative dot plot of FoxP3 and CD25 expression on CD3^+^ CD4^+^ T cells in NSCLC tumor. Percentages of FoxP3^+^ and FoxP3^−^ T cells among CD3^+^ CD4^+^ CD25^bright^ T cells are indicated in the appropriate gates. **(B)** Representative dot plots of CD25 expression on CD3^+^ CD4^+^ T cells in a TLS-B^low^ tumor (left panel) and a TLS-B^high^ tumor (right panel). Percentages of CD4^+^ CD25^bright^ Tregs are indicated in the appropriate gate. **(C)** Representative dot plots of FoxP3 expression on total CD3^+^ CD4^+^ T cells in a TLS-B^low^ tumor (left panel) and a TLS-B^high^ tumor (right panel). Percentages of CD4^+^ FoxP3^+^ Tregs are indicated in the appropriate gate. **(D)** Graph represents the frequencies of FoxP3^bright^ cells among total TIL CD3^+^ CD4^+^ T cells with tumors stratified into TLS-B^low^ and TLS-B^high^ groups. Medians are indicated by horizontal red lines. *P*-values were calculated with Mann Whitney test. *p < 0.05. Rectangles identify tumors with high or low frequencies of CD4^+^ Tregs in both groups. **(E)** Relative percentages of tumors with high (black) or low (white) frequencies of CD4^+^ Tregs in TLS-B^low^ and TLS-B^high^ groups. NSCLC, non-small-cell lung cancer; TIL, tumor-infiltrating lymphocyte; TLS, tertiary lymphoid structure.

Because CD4^+^ Tregs strongly express CD25 (together with GITR and Tim-3) (18), we further characterized the phenotype of CD25^+^ TIL T cells relative to TLS-B cell density. We detected a population of CD25^bright^ CD4^+^ T cells co-expressing FoxP3 (Figure 5A) that was more frequent among total TIL CD4^+^ T cells in TLS-B^low^ *vs*. TLS-B^high^ tumors (Figures 5B–E). A significant negative correlation was observed between the geomean of CD25 expressed by CD4^+^ T cells and TLS-B cell density in the corresponding tumor sample (Supplementary Figure 9A), and to a lesser extent, between the percentage of CD25^bright^ CD4^+^ T cells and TLS-B cell density (Supplementary Figure 9B). The percentage of Tregs among total CD4^+^ TIL T cells was higher in TLS-B^low^ tumors than TLS-B^high^ tumors (Figure 5D). This observation was confirmed on FFPE tumor sections, where 50% of TLS-B^low^ tumors were CD4^+^ Treg^high^, compared with 8% in TLS-B^high^ tumors (Figure 5E).

These results demonstrate that Tregs are most frequent in the CD4^+^ T cell compartment of tumors with a low density of TLS-B cells.

### The Combination of TLS-B Cell and Treg Densities Is a Strong Prognostic Indicator of Clinical Outcome in NSCLC Patients

**Figure 6:**
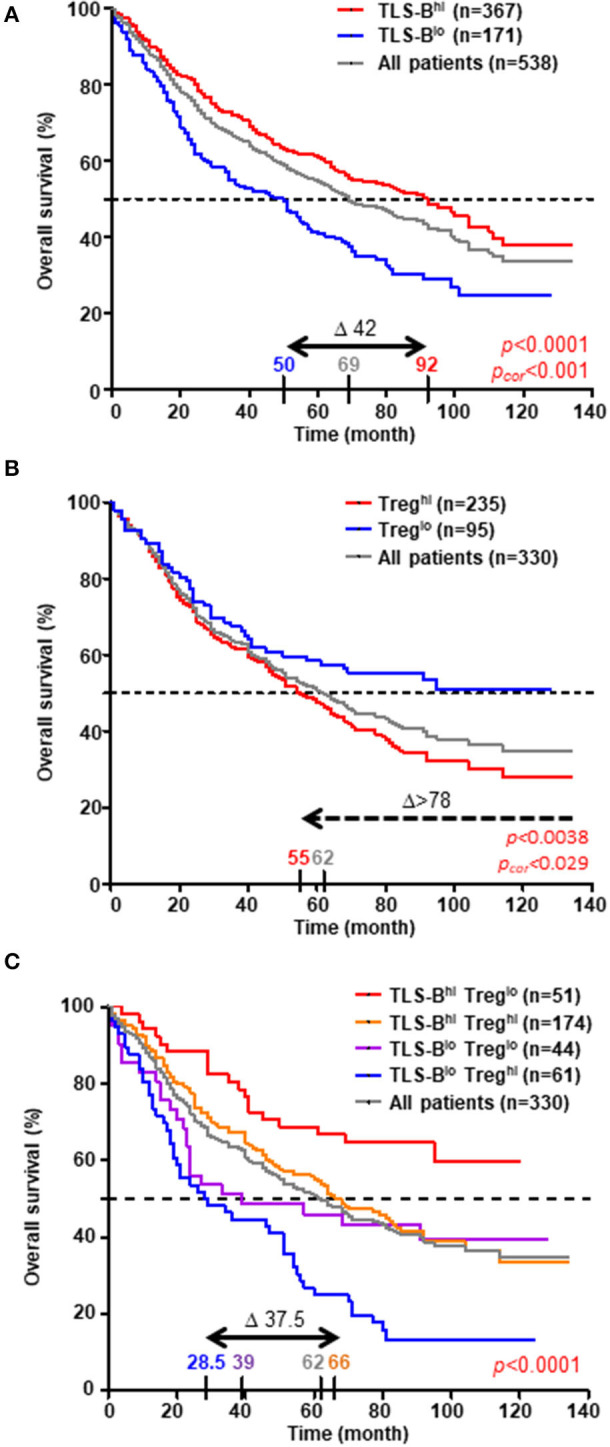
High density of TLS-B cells cancels out the negative impact of high Treg density on overall survival. Kaplan-Meier curves of overall survival (OS) among the retrospective cohort (**A**, *n* = 538 NSCLC patients; **B,C**, *n* = 330 NSCLC patients), according to **(A)** CD20^+^ TLS-B cell density, **(B)** CD3^+^ FoxP3^+^ Treg density, and **(C)** combined CD20^+^ TLS-B cell and CD3^+^ FoxP3^+^ Treg densities (optimal cut-off value). The horizontal dashed line on each graph represents the median survival. Median survival values for each group of patients are also reported on the graph, as well as the difference in months between the best and worst surviving groups (Δ). *P*-values were determined with the log-rank test and corrected when indicated according to the formula proposed by Altman *et al*. (15).

The prognostic value of CD20^+^ TLS-B cells was next investigated in a retrospective cohort (*n* = 538 NSCLC, Supplementary Table 1 and Supplementary Figures 1B–D), through two distinct methods. We first confirmed that high TLS-B cell density was associated with prolonged survival [Figure 6A, by applying the optimal cut-off determined in Supplementary Figure 3A; Supplementary Figure 10, with the median (2, 12)]. The median values for TLS-B cell density did not differ significantly by tumor stage (Supplementary Figure 11A) or histological subtype (Supplementary Figure 12A). However, its favorable impact on survival was observed as early as stage I (Δ between best and worst median survival = 24 months, *p* = 0.005), peaked at stage II (Δ between best and worst median survivals = 76 months, *p* = 0.0007) but no longer existed at stages III-IV (Δ between best and worst median survival = 7 months, *p* = 0.1649) (Supplementary Figures 11B–D). The prognostic value of TLS-B cell density was consistent for the ADC and SCC subtypes (Δ between the best and worst median survival = around 50 months, Supplementary Figures 12B,C).

Next, we evaluated the prognostic value of CD3^+^ FoxP3^+^ Tregs (*n* = 330 patients, using the optimal cut-off determined in Supplementary Figure 3B). High Treg density was associated with a poor clinical outcome (Δ between best and worst median survival > 78 months, Figure 6B). Moreover, the combination of TLS-B cells and Tregs improved OS prediction over each individual parameter, identifying a group of NSCLC patients with the best clinical outcome (TLS-B^high^/Treg^low^, median survival still unreached at 120 months, Figure 6C). High TLS-B cell density was even able to counterbalance the deleterious impact of high Treg density on patient survival (median OS = 66 months for TLS-B^high^/Treg^high^ patients *vs*. 28.5 months for TLS-B^low^/Treg^high^ patients).

In univariate Cox regression analysis, patients' age (HR = 2.239), pTNM stage (HR = 2.841), and high Treg density (HR = 1.617) were each significantly associated with poor OS, and high TLS-B cell density (HR = 0.606) with long-term OS (Table 1). Multivariate Cox analysis showed that patients' age, pTNM stage, TLS-B cell density, and Tregs were independent prognostic factors (Table 2).

**Table 1:** Prognostic parameters for overall survival of NSCLC patients in univariate analysis.

**Variable**	**Class**	**Hazard ratio**	**95% CI**	***P*-value**
Gender	Female	1		
	Male	1.268	0.958-1.677	0.097
Age	Year	2.239	1.406-3.565	**0.00069**
	<60	1		
	≥60	1.283	1.014-1.624	**0.038**
Histological subtype	ADC	1		
	SCC	1.090	0.846-1.406	0.50
	Others	1.554	1.009-2.393	**0.045**
pTNM stage	I			
	II	1.349	1.012-1.797	**0.041**
	III-IV	2.841	2.190-3.685	**3.77e-15**
Smoking history	Pack-Year	1.027	0.960-1.1	0.44
	<15	1		
	≥15	1.193	0.835-1.707	0.33
TLS-B cell density	Log	0.711	0.599-0.845	**0.00010**
	TLS-B^low^	1		
	TLS-B^high^	0.606	0.482-0.761	**1.67e-05**
Treg density	Log	1.071	0.983-1.166	0.1157
	Treg^low^	1		
	Treg^high^	1.617	1.164-2.246	**0.00420**

*The Cox regression model was used for univariate analysis. A p-value < 0.05 was considered statistically significant (bold values). N = 538 patients for CD20^+^ TLS-B cell density, n = 330 patients for CD3^+^ FoxP3^+^ Treg density. ADC, adenocarcinoma; SCC, squamous cell carcinoma; TLS, tertiary lymphoid structure*.

**Table 2:** Multivariate Cox proportional hazards analysis for overall survival of NSCLC patients.

**Variable**	**Class**	**Hazard ratio**	**95% CI**	***P*-value**
Age	Year	1.814	1.038-3.171	**0.0366**
pTNM stage	I			
	II	1.291	0.906-1.840	0.1578
	III-IV	2.620	1.899-3.614	**4.47e-09**
TLS-B cell density	Log	0.618	0.496-0.770	**1.80e-05**
Treg density	Log	1.119	1.025-1.222	**0.0119**

*The Cox regression model was used for multivariate analysis. Parameters identified in univariate analysis as possibly influencing the clinical outcome (p < 0.05) were introduced into a multivariate Cox-proportional hazards regression model. A p < 0.05 was considered statistically significant (bold values). N = 330 NSCLC patients*.

Taken together, these data show that high TLS-B cell density significantly and positively affects survival of NSCLC patients, and its evaluation makes it possible to better stratify patient prognosis, especially when combined with low Treg cell density.

## Discussion

The presence of TLS in the tumor microenvironment favorably affects prognosis for many cancers (4, 9–11). Various factors have been proposed to explain its positive prognostic value, and more recently, its predictive value for ICP responsiveness (2, 9–11). Nonetheless the ability of TLS, and especially TLS-B cells, to modulate T cell activation, co-stimulatory properties, and exhaustion remains poorly understood. Among the numerous cellular mechanisms that can take place in TLS is the presentation by B cells of antigen to CD4^+^ T cells (17–19).

In this study, we consistently observed high expression of both HLA-DR and CD40 by most tumor-infiltrating B cells, as well as CD80, CD83, and CD86 expression by a subset of B cells. These findings indicate that the tumor microenvironment does not impair the capacity of B cells to present peptide antigens by MHC-class II molecules through loss of MHC-class II expression. Further investigation showed that TLS-B density was positively correlated with a specific CD4^+^ T cell signature, including *POU2AF1*, a gene encoding the transcriptional co-activator OCA-B (also called Bob1 or OBF-1) that directly contributes to IFN-γ and IL-2 promoter activities (16) and which is mandatory for the *in vivo* generation of CD4^+^ memory T cells (20). These findings are consistent with the positive impact of TLS-B cells on CD4^+^ T cell clonality that we previously reported in NSCLC (10).

Among B cell subsets, higher frequencies of activated CD69^+^ and CD86^+^ B cells, together with lower frequencies of ICOS-L^+^ B cells, reflect favorable B cell/T cell interactions, mutual activation, and antigen presentation within TLS. This result is consistent with ICOS-L membrane expression, which is known to be downregulated on B cells after interaction with ICOS on T cells (21). On the T cell side, we observed higher frequencies in NSCLC tumors of ICOS^+^, 4-1BB^+^, GITR^+^, and OX40^+^ CD4^+^ T cells. All of these molecules belong to the Tumor Necrosis Factor Receptor (TNF-R) co-signaling receptor family. After activation through a productive TCR-MHC-peptide interaction, 4-1BB, OX40, and GITR are induced and synergize with the TCR and CD3 molecules to promote cell cycle progression, survival, and cytokine production by T cells (22). OX40 and 4-1BB are also both important in the generation of antigen-specific memory T cells. In particular, OX40 plays a key role in the establishment of a robust CD4^+^ memory T cell response (23).

Exhausted T cells are characterized by the expression of several ICPs, in particular, Tim-3, a common determinant of exhaustion. ICPs are now the targets of several blocking monoclonal antibodies that improve survival among a substantial number of cancer patients (24, 25). Our observation, consistent with the literature, of higher percentages of T cells expressing PD-1, Tim-3, and TIGIT, as well as of fully exhausted [PD-1^+^ Tim-3^+^ TIGIT^+^] triple-positive T cells, in the CD4^+^ T cell compartment in tumors compared with distant NT sites underlines the interest in targeting these three ICPs. The strongest and most significant negative correlation, however, was observed between Tim-3^+^ CD4^+^ T cells and TLS-B density. Furthermore, the frequency of [PD-1^+^ TIGIT^+^] CD4^+^ T cells was positively correlated with TLS-B density, which again suggests that TLS-B cells may promote CD4^+^ T cell activation while limiting their exhaustion (19, 22). Accordingly, these results not only confirm the relevance of multiple ICP blockades in NSCLC, but also suggest that favoring lymphoid neogenesis in tumors might be a powerful alternative mechanism for promoting activation and ultimately anti-tumor immunity.

In line with these results, high TLS-B density was also negatively correlated with a CD4^+^ T cell compartment expressing genes and molecules associated with Treg phenotype, including CD5, CD25, GITR, and Tim-3. Henderson *et al*. recently demonstrated that CD5 plays a major role in promoting Treg cell development by inhibiting cytokine receptor-mediated activation of mTOR, a regulator of Foxp3 induction (17). Similarly, GITR and Tim-3 are both highly expressed on CD4^+^ Tregs (26, 27), and anti-GITR antibodies are currently being developed to target deleterious Tregs in cancers (28). Reconsidering this regulatory T cell population with the FoxP3 marker, we again observed a higher percentage of CD4^+^ FoxP3^+^ Tregs in tumors from TLS-B^low^ compared with TLS-B^high^ patients. Moreover, Treg^high^ tumors were observed mainly among the group of TLS-B^low^ tumors. Two studies in atherosclerosis (29) and allo-engraftment (30) have reported that TLS can temper inflammation or favor allograft acceptance by promoting Treg differentiation. In secondary lymphoid organs, fibroblastic reticular cells can present self-antigens and promote tolerance rather than immune activation (31, 32), partly by promoting Treg differentiation (33, 34). In the present study, TLS-B cell density correlated negatively with CD4^+^ T cells expressing dipeptidyl peptidase-4 (*DPP4*), which inhibits the proliferative capacity of tumor-infiltrating effector T cells when expressed by cancer-associated fibroblast-subset 1 (CAF-S1) conditioned-Treg in triple-negative breast cancer (35). We might speculate that the origin and functions of CAF play a pivotal role in the tumor microenvironment. In particular, some CAF may be critical for TLS neogenesis as lymphoid inducer cells, as well as for TLS immune function. The depletion of Tregs in different tumor models has been associated with lower tumor growth and with the development of high endothelial venules (HEV)—the only category of blood vessels correlated with a favorable clinical outcome in malignancies; they also play a major role in the recruitment of circulating immune cells into both SLO and TLS (6). The association of Treg depletion with increased CD4^+^ and CD8^+^ T cell infiltration and TLS formation in carcinogen-induced tumors and lung adenocarcinoma models (36, 37) also demonstrates cross-talk between Tregs and TLS.

The transfer of Foxp3^+^ CD4^+^ Tregs enables the formation of GC in Peyer's patches of T cell deficient mice. After transfer, these Tregs lose their FoxP3 expression and migrate into B-cell follicles, in which they differentiate into T_FH_ cells on CD40/CD40L-dependent interaction with B cells and consistent with the detection of T_FH_ in breast cancer- and NSCLC-associated TLS (38–40). This could explain how the presence of TLS-B cells within the tumor microenvironment is able to reverse the deleterious impact of Tregs on patient survival.

Our detailed analysis of the co-receptors and ICPs expressed by CD4^+^ T cells in NSCLC tumors paves the way for innovative therapeutic strategies. In mouse tumor models, agonist antibodies to TNF-R induced a marked increase of antigen-specific CD8^+^ and CD4^+^ T cell responses and generated memory T cells (41–43). Many current clinical studies are looking at immune-stimulatory antibodies, including 4-1BB, CD27, OX40, GITR, and CD40 agonists, for several oncologic indications, as standalone treatment or in combination with ICP inhibitors targeting the PD-1/PD-L1 axis. Because only a fraction of patients achieve long-term remission with a single ICP blockade, these combinations are highly interesting, especially given the preclinical evidence of a synergistic effect against tumor progression (44, 45). The phenotype of intratumor CD4^+^ T cells thus suggests that 4-1BB may represent an excellent target for future combined immunotherapies in NSCLC (25, 45).

Overall, our results provide new key elements for understanding the role that TLS-B cells play in NSCLC survival. They also reveal the relationship between TLS-B cells and CD4^+^ T cell infiltrate. We can hypothesize that TLS-B cells positively modulate anti-tumor T cell immunity by limiting CD4^+^ Treg generation, while favoring CD4^+^ T cell activation and restraining CD4^+^ T cell exhaustion. Our data are consistent with the relationship observed between TLS-B cell density and ICP response in patients with metastatic melanoma, RCC and sarcoma (9–11). They also provide a detailed picture of the various co-stimulatory molecules and immune checkpoints expressed by tumor-infiltrating CD4^+^ T cells and demonstrate the promise of new combined cancer immunotherapies to be explored in NSCLC patients.

## Data Availability Statement

Data are available upon reasonable request. All data relevant to the study are included in the article or uploaded as supplementary information. All data generated that are relevant to the results presented in this article are included in this article. Other data that were not relevant for the results presented here are available from the corresponding author, M-CD-N, upon reasonable request.

## Ethics Statement

The studies involving human participants were reviewed and approved by CPP (n° 2008-133, 2012-0612 and 2017-A03081-52). Samples from healthy volunteers were obtained at the Etablissement Français du Sang (EFS, Paris, France, n° 15EFS012 and n° 18EFS033). The patients/participants provided their written informed consent to participate in this study.

## Author Contributions

M-CD-N and SH conceptualized the research. CG, PD-M, SK, JB, HK, LL, JG, and FL performed research investigation. AS-G, DG, NG, PV, ML, DD, and MA provided resources (fresh human specimens and patient enrollment). CG and M-CD-N performed formal analysis of the data. CG wrote the initial draft. M-CD-N, KS, SH, and J-LT revised and edited the manuscript. M-CD-N performed funding acquisition. All authors read and approved the final version of the manuscript.

## Conflict of Interest

DG and AS-G received fees from Medtronic company for presentations not in relation with this topic. DG is consultant for an instrument manufacturer (Delacroix Chevalier). KS and SH are full-time employees of AstraZeneca. The remaining authors declare that the research was conducted in the absence of any commercial or financial relationships that could be construed as a potential conflict of interest.

## Supplementary Material

Supplementary Figure 1 Presence of tumor-infiltrating CD20^+^ TLS-B cells and CD3^+^ FoxP3^+^ T cells in NSCLC patients. HES **(A)** and CD20/CD21 **(B)**, CD20/pan-Cytokeratins **(C)** and CD3/FoxP3 **(D)** double IHC staining of FFPE lung tumor sections of NSCLC patients. Magnifications: **(A,B)** x100, **(C,D)** x200. FFPE, formalin-fixed paraffin-embedded; IHC, immunohistochemical; NSCLC, non-small-cell lung cancer.

Supplementary Figure 2 Flow cytometry gating strategy. Gating strategy used for flow cytometry analysis to evaluate the expression of activation markers, co-stimulatory molecules and immune checkpoints on CD19^+^ B cells (gate 1) and CD3^+^ CD4^+^ T cells (gate 3) of NSCLC patients and healthy donors. Illustration of the different B cell subsets based on the differential expression of IgD and CD38 (naïve IgD^+^ CD38^+/−^ B cells, transitional IgD^+^ CD38^+^ B cells (Transi.), germinal center (GC) IgD^−^ CD38^+^ B cells, memory (Mem.) IgD^−^ CD38^+/−^ B cells, and IgD^−^ CD38^++^ plasma cells (PC). Discrimination of the different CD4^+^ T cell subsets based on the differential expression of CD45RA and CCR7 (naïve CD45RA^+^ CCR7^+^ T cells, central-memory (CM) CD45RA^−^ CCR7^+^ T cells, effector-memory (EM) CD45RA^−^ CCR7^−^ T cells, terminally differentiated CD45RA^+^ (EMRA) CCR7^−^ effector T cells. CM, central-memory; EM, effector-memory; EMRA, terminally differentiated CD45RA^+^; GC, germinal center; Mem, memory; NSCLC, non-small-cell lung cancer; PC, plasma cell; Transi, transitional.

Supplementary Figure 3 Determination of the optimal cut-off *p*-values to discriminate between high and low densities of intratumor TLS-B cells and Tregs. Log-rank *P-*values for overall survival according to CD20^+^ TLS-B cell **(A)** and CD3^+^ FoxP3^+^ Treg **(B)** densities. The horizontal gray dashed line indicates the limit of significance of the *p*-value (*p* = 0.05). The vertical black line indicates the selected cut-off *p*-value that was used to discriminate between high and low density groups. The vertical black dashed lines indicate the values excluded by Altman's formula (15). Optimal cut-off *p*-values are 0.3256612% of total tumor surface for CD20^+^ TLS-B cell density, and 21.93277 cells/mm^2^ for CD3^+^ FoxP3^+^ Treg density.

Supplementary Figure 4 Correlation between genes expressed by TIL CD4^+^ T cells and TLS-B cell density. **(A)** Correlation between gene expression by sorted TIL CD4^+^ T cells and TLS-B cell density. Genes significantly correlated with TLS-B cell density (0.05 < FDR < 0.10 and 0.10 < FDR < 0.15) are shown. Vertical dashed lines represent limits for correlation factors (< -0.5 and >+0.5). Statistical test used: Spearman test. **(B)** Normalized mean counts of genes positively or negatively (FDR < 0.15) correlated with TLS-B cell density are shown. Mean ± SEM of normalized counts. FDR, false discovery rate; TIL, tumor-infiltrating lymphocyte.

Supplementary Figure 5 Memory B cells and plasma cells are more frequent in tumors than at non-tumor sites. **(A)** Representative dot plot of B cell subsets among total CD19^+^ B cells in NSCLC tumor, based on the differential expression of IgD and CD38 (IgD^+^ CD38^+/−^ naïve B cells; IgD^+^ CD38^+^ pre-GC B cells; IgD^−^ CD38^+^ GC-B cells; IgD^−^ CD38^+/−^ memory B cells; IgD^−^ CD38^++^ PC). **(B)** Histogram represents the percentages of each B cell subset among total CD19^+^ B cells in NSCLC tumor, NT lung, NSCLC peripheral blood and healthy donor peripheral blood (mean ± SEM) sites. *P*-values were calculated with one-way ANOVA/Kruskal-Wallis/Dunn's test. **(C)** Graphs represent the percentages of each B cell subset among total B cells, in each compartment of NSCLC patients. *P*-values were calculated with one-way ANOVA/Friedman/Dunn's test. ^*^*P* < 0.05, ^**^*P* < 0.005, ^***^*P* < 0.001. GC, germinal center; HD, healthy donor; NSCLC, non-small-cell lung cancer; NT, non-tumor; pre-GC, pre-germinal center; PC, plasma cell.

Supplementary Figure 6 Analysis of B cell subsets in different anatomical sites. **(A)** Representative dot plots of expression of CD27/CD38 (upper panels), CD69/IgD (middle panels), and ICOS-L/IgD (lower panels) among total CD19^+^ B cells in NSCLC tumors (left panels) or NSCLC peripheral blood and lymph nodes. Percentages of cells are indicated in each quadrant. **(B)** Frequencies of CD86^+^ cells among each B cell subset in NSCLC tumors. Means are indicated by horizontal red lines. *P*-values were calculated with one-way ANOVA/Kruskal-Wallis/Dunn's test. ^***^*p* < 0.001. **(C)** Representative dot plot of regulatory B cells (Bregs) among total CD19^+^ B cells from NSCLC tumor (upper panel), non-tumor lung (lower left panel) or NSCLC peripheral blood (lower right panel) sites, based on the differential expression of CD38 and CD24. Percentages of CD38^high^ CD24^high^ Bregs are indicated in the corresponding gate. GC, germinal center; LN, lymph node; NSCLC, non-small-cell lung cancer; pre-GC, pre-Germinal Center; NT, non-tumor; PC, plasma cell.

Supplementary Figure 7 Tumors have higher frequencies of effector-memory CD4^+^ T cells than non-tumor cells. **(A)** Representative dot plot of T cell subsets among total CD3^+^ CD4^+^ T cells in NSCLC tumors, based on the differential expression of CD45RA and CCR7 (CD45RA^+^ CCR7^+^ naïve T cells; CD45RA^−^ CCR7^+^ central-memory (CM) T cells; CD45RA^−^ CCR7^−^ effector-memory (EM) T cells; CD45RA^+^ CCR7^−^ terminally differentiated CD45RA^+^ effector cells (TEMRA)). **(B)** Histogram represents the percentages of each T cell subset among total CD3^+^ CD4^+^ T cells from NSCLC tumor, non-tumor lung (NT), NSCLC peripheral blood and healthy donor (HD) peripheral blood (mean ± SEM) sites. *P*-values were calculated with one-way ANOVA/Kruskal-Wallis/Dunn's test. ^*^*p* < 0.05, ^**^*p* < 0.005, ^***^*p* < 0.001. CM, central-memory; EM, effector-memory; HD, healthy donor; NSCLC, non-small-cell lung cancer; NT, non-tumor; TEMRA, terminally differentiated CD45RA^+^ cell.

Supplementary Figure 8 Correlation between TLS-B cell density and specific CD4^+^ T cell markers in tumors. Graphs represent the frequencies of cells expressing the mentioned markers among TIL CD3^+^ CD4^+^ T cells with tumors stratified into TLS-B^Low^ (*n* = 7, first quartile) and TLS-B^High^ (*n* = 7, last quartile) groups. Means are indicated by horizontal red lines. *P*-values were calculated with the Mann Whitney test. ^*^*p* < 0.05. ns, non-significant.

Supplementary Figure 9 Lower percentages of CD25^bright^ cells/total CD4^+^ T cells in TLS-B^high^ vs. TLS-B^low^ NSCLC tumors. Correlations between **(A)** CD25 geomean on intratumor CD3^+^ CD4^+^ T cells (*n* = 20) or **(B)** the percentage of CD25^brigh^ T cells among intratumor CD3^+^ CD4^+^ T cells (*n* = 20) with TLS-B cell density in the corresponding tumor sections. Statistical significance was determined by the Spearman test.

Supplementary Figure 10 High density of TLS-B cells is associated with better overall survival of NSCLC patients. Kaplan-Meier curves of overall survival (OS) among the retrospective cohort (*n* = 538 patients) according to TLS-B cell density (using median cut-off value). The horizontal dashed line on each graph represents the median survival. Median survival values for each group of patients are also reported on the graph, as well as the difference in months between the best and worst surviving groups (Δ). *P*-value was determined using the log-rank test.

Supplementary Figure 11 No differences in TLS-B cell density between NSCLC tumor stages but prognostic value of TLS-B cell density best in stages I and II. **(A)** Graph represents the distribution of TLS-B cell densities for different NSCLC stages in the retrospective cohort (*n* = 538). Median density in each group is indicated by red horizontal lines. Statistical tests used: one-way ANOVA, Kruskal-Wallis test, Dunn's Multiple Comparison. **(B–D)** Kaplan-Meier overall survival (OS) curves by TLS-B cell density among NSCLC **(B)** stage I (*n* = 238), **(C)** stage II (*n* = 146), or **(D)** combined stages III-IV (*n* = 154). Optimal cut-off values used were determined in the whole retrospective cohort. The horizontal dashed line on each graph represents the median survival. Median survival values for each group of patients are also reported on the graph, as well as the difference in months between the best and worst surviving group (Δ). *P*-values were determined with the log-rank test.

Supplementary Figure 12 Densities and prognostic value of TLS-B cells according to histological subtypes. **(A)** Graph represents the distribution of TLS-B cell densities among different NSCLC histological subtypes in the retrospective cohort. Median densities in each group are indicated by red horizontal lines. Statistical tests used: one-way ANOVA, Kruskal-Wallis test, Dunn's Multiple Comparison. **(B,C)** Kaplan-Meier overall survival (OS) curves by TLS-B cell density in patients with **(B)** adenocarcinoma (ADC, *n* = 195) or **(C)** squamous cell carcinoma (SCC, *n* = 141) NSCLC subtype. The optimal cut-off values used were determined from the entire retrospective cohort (*n* = 538). The horizontal dashed line on each graph represents the median survival. Median survival values for each group of patients are also reported on the graph, as well as the difference in months between the groups with the best and worst survival (Δ). *P*-values were determined with the log-rank test. ADC, adenocarcinoma; LCC, large cell carcinoma; LCNEC, large cell neuroendocrine carcinoma; SCC, squamous cell carcinoma.

Supplementary Table 1 Clinical and pathological features of 538 NSCLC patients enrolled in the retrospective study. Pathologic staging of lung cancer was determined according to the new TNM staging classification (46). Histological subtypes were determined according to the WHO classification (47). Patients received neither preoperative chemotherapy nor radiotherapy. Patients with mixed histologic features, a T3 tumor, or pleural invasion were ineligible. Others: large cell carcinoma (4%), sarcomatoid carcinoma (1.5%), and large cell neuroendocrine carcinoma (0.5%). ND, not determined.

Supplementary Table 2 Clinical and pathological features of the 56 NSCLC patients enrolled in the prospective study. Pathologic staging of lung cancer was determined according to the new TNM staging classification (46). Histological subtypes were determined according to the WHO classification (47). Patients received neither pre-operative chemotherapy nor radiotherapy. Patients with mixed histologic features, a T3 tumor, or pleural invasion were ineligible. Others: large cell carcinoma (1.5%) and large cell neuroendocrine carcinoma (1.5%). ND, not determined.

Supplementary Table 3 Antibodies and reagents used for immunohistochemistry and immunofluorescence analyses. Stainings were performed on FFPE NSCLC samples. pH 6.0, heat-induced epitope retrieval in 10 mM Citrate buffer pH 6.0; pH 8.0, heat-induced epitope retrieval in 0.1 mM EDTA buffer pH 8.0; TRS, citrate pH 6.0-like solution (Dako-Agilent). AEC, 3-amino-9-ethylcarbazole substrate; AP, alkaline phosphatase; APAAP, alkaline phosphatase anti-alkaline phosphatase (AP-conjugated anti-AP mouse antibody); HRP, horseradish peroxidase; NA, Not Applicable; SAP, substrate for alkaline phosphatase; TRS, target retrieval solution; UC, uncoupled (antibody).

Supplementary Table 4 Antibodies and reagents used for flow cytometry analyses and flow cytometry-based cell sorting experiments. NA, Not Applicable.

## Abbreviations

ADC: adenocarcinoma
Breg: regulatory B cell
CAF: cancer-associated fibroblast
CM: central-memory
DC: dendritic cell
EM: effector-memory
FDR: false discovery rate
FFPE: formalin-fixed paraffin-embedded
GC: germinal center
HD: healthy donor
HEV: high endothelial venule
ICP: immune checkpoint
IF: immunofluorescence
IHC: immunohistochemistry
LN: lymph node
mAb: monoclonal antibody
NSCLC: non-small cell lung cancer
NT: non-tumor
P_cor_: corrected P-value
PC: plasma cell
SCC: squamous cell carcinoma
SLO: secondary lymphoid organ
TEMRA: effector-memory CD45RA^+^ T cell
T_FH_: follicular-helper T cell
TIL: tumor-infiltrating lymphocyte
TLS: tertiary lymphoid structure
TNF-R: tumor necrosis factor receptor
Treg: regulatory T cell.
