# Supplementary figures and images for: Tertiary Lymphoid Structure-B Cells Narrow Regulatory T Cells Impact in Lung Cancer Patients
Source: Front Immunol. 2021 Mar 8;12:626776. doi: 10.3389/fimmu.2021.626776 (PMC7983944; doi:10.3389/fimmu.2021.626776)

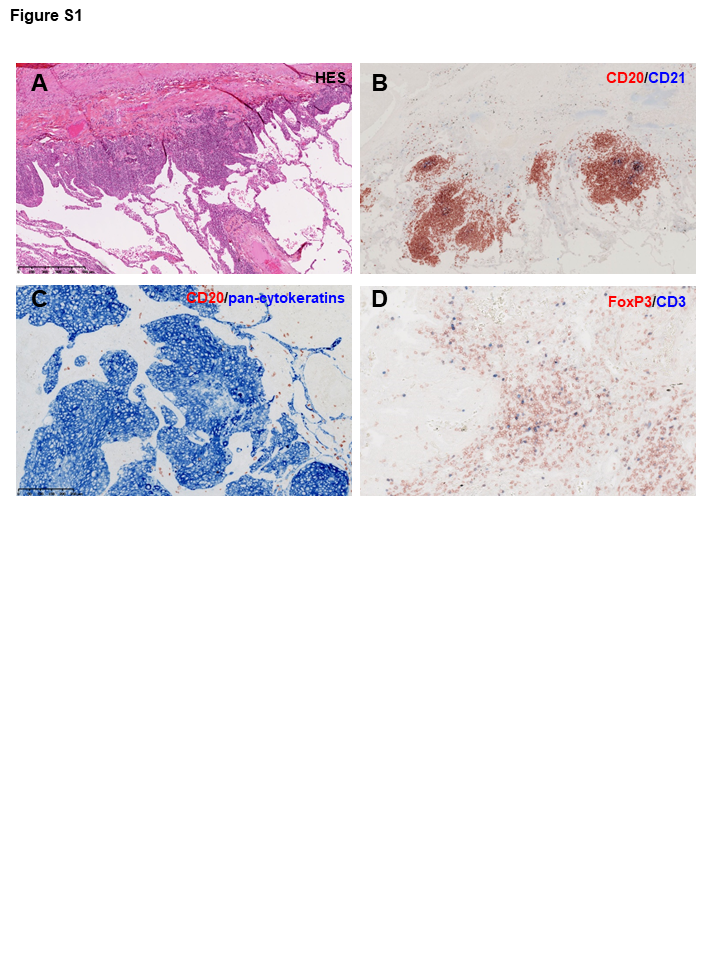

Supplement: Supplementary Figure 1 — Presence of tumor-infiltrating CD20+ TLS-B cells and CD3+ FoxP3+ T cells in NSCLC patients. HES (A) and CD20/CD21 (B), CD20/pan-Cytokeratins (C) and CD3/FoxP3 (D) double IHC staining of FFPE lung tumor sections of NSCLC patients. Magnifications: (A,B) x100, (C,D) x200. FFPE, formalin-fixed paraffin-embedded; IHC, immunohistochemical; NSCLC, non-small-cell lung cancer. [file Image_1.tif]

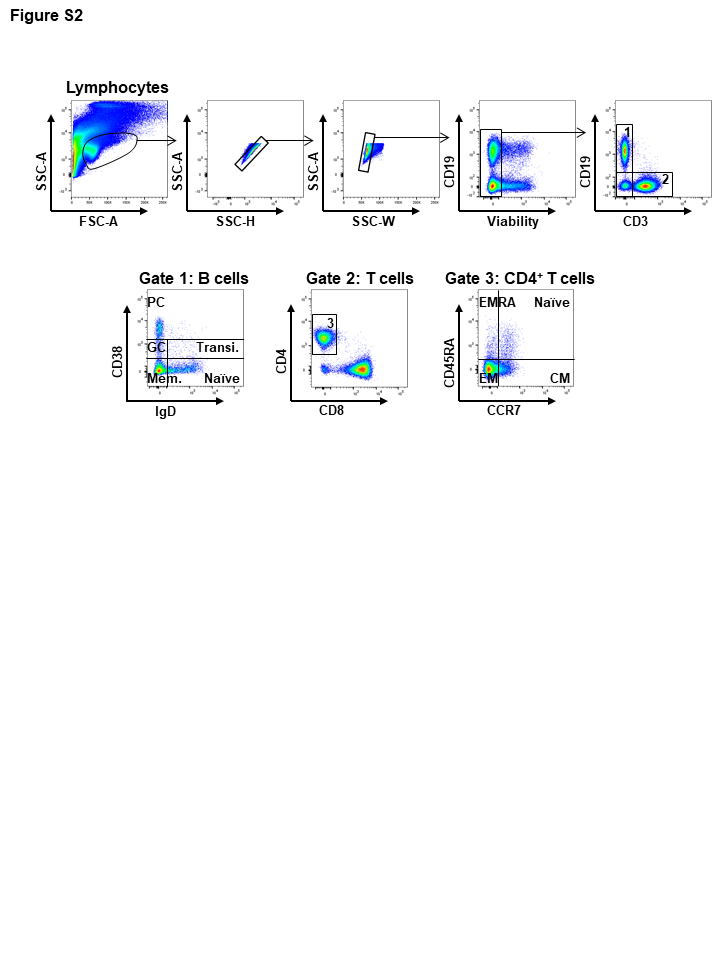

Supplement: Supplementary Figure 2 — Flow cytometry gating strategy. Gating strategy used for flow cytometry analysis to evaluate the expression of activation markers, co-stimulatory molecules and immune checkpoints on CD19+ B cells (gate 1) and CD3+ CD4+ T cells (gate 3) of NSCLC patients and healthy donors. Illustration of the different B cell subsets based on the differential expression of IgD and CD38 (naïve IgD+ CD38+/− B cells, transitional IgD+ CD38+ B cells (Transi.), germinal center (GC) IgD− CD38+ B cells, memory (Mem.) IgD− CD38+/− B cells, and IgD− CD38++ plasma cells (PC). Discrimination of the different CD4+ T cell subsets based on the differential expression of CD45RA and CCR7 (naïve CD45RA+ CCR7+ T cells, central-memory (CM) CD45RA− CCR7+ T cells, effector-memory (EM) CD45RA− CCR7− T cells, terminally differentiated CD45RA+ (EMRA) CCR7− effector T cells. CM, central-memory; EM, effector-memory; EMRA, terminally differentiated CD45RA+; GC, germinal center; Mem, memory; NSCLC, non-small-cell lung cancer; PC, plasma cell; Transi, transitional. [file Image_2.tif]

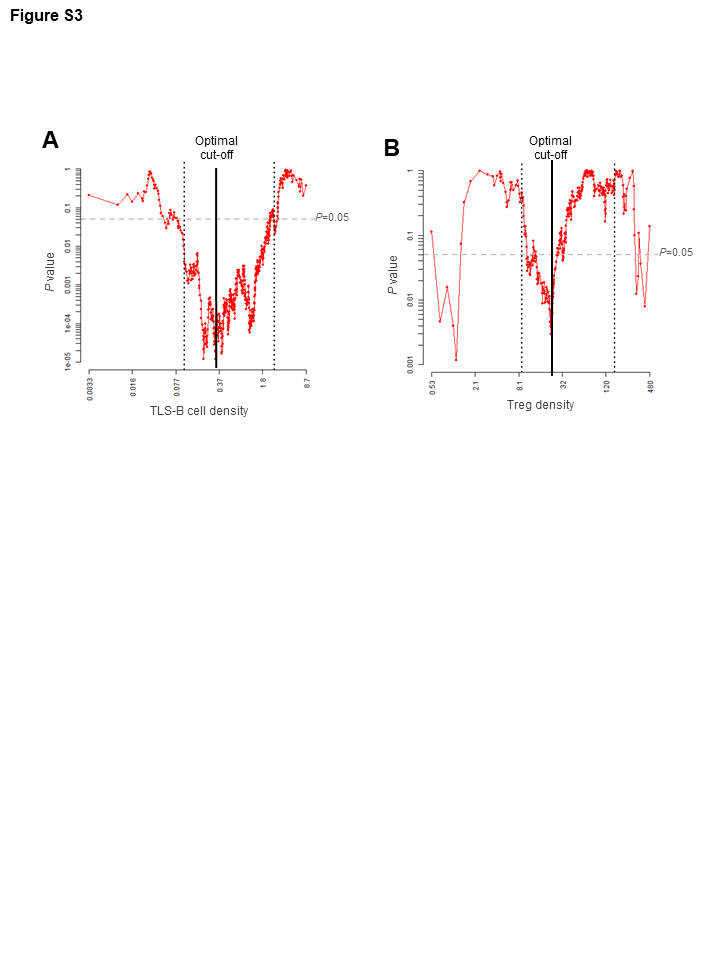

Supplement: Supplementary Figure 3 — Determination of the optimal cut-off p-values to discriminate between high and low densities of intratumor TLS-B cells and Tregs. Log-rank P-values for overall survival according to CD20+ TLS-B cell (A) and CD3+ FoxP3+ Treg (B) densities. The horizontal gray dashed line indicates the limit of significance of the p-value (p = 0.05). The vertical black line indicates the selected cut-off p-value that was used to discriminate between high and low density groups. The vertical black dashed lines indicate the values excluded by Altman's formula (15). Optimal cut-off p-values are 0.3256612% of total tumor surface for CD20+ TLS-B cell density, and 21.93277 cells/mm2 for CD3+ FoxP3+ Treg density. [file Image_3.tif]

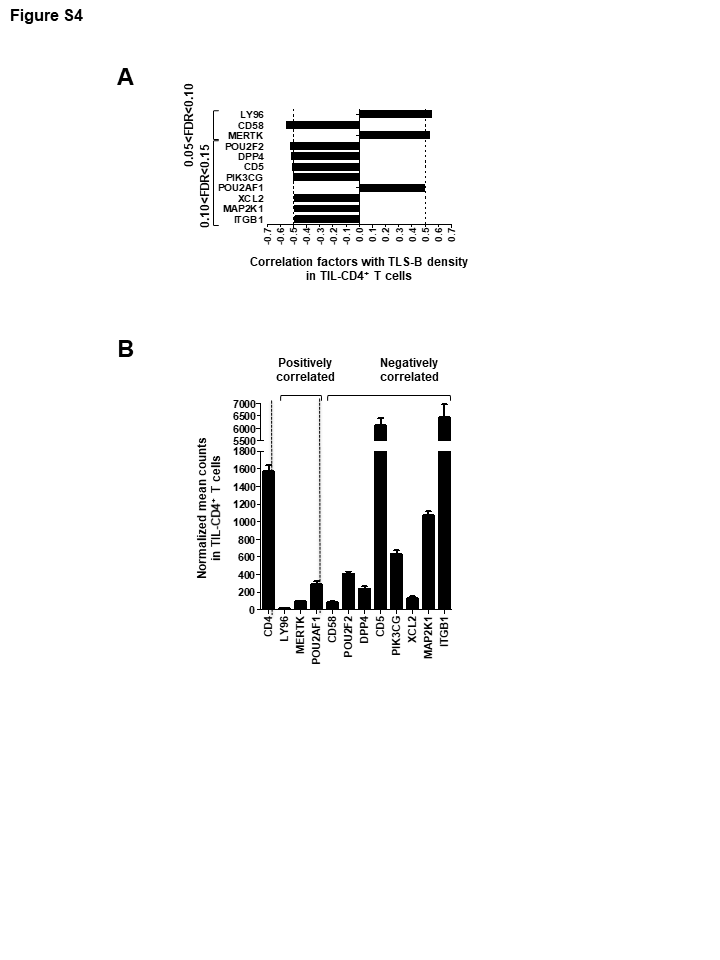

Supplement: Supplementary Figure 4 — Correlation between genes expressed by TIL CD4+ T cells and TLS-B cell density. (A) Correlation between gene expression by sorted TIL CD4+ T cells and TLS-B cell density. Genes significantly correlated with TLS-B cell density (0.05 < FDR < 0.10 and 0.10 < FDR < 0.15) are shown. Vertical dashed lines represent limits for correlation factors (< -0.5 and >+0.5). Statistical test used: Spearman test. (B) Normalized mean counts of genes positively or negatively (FDR < 0.15) correlated with TLS-B cell density are shown. Mean ± SEM of normalized counts. FDR, false discovery rate; TIL, tumor-infiltrating lymphocyte. [file Image_4.tif]

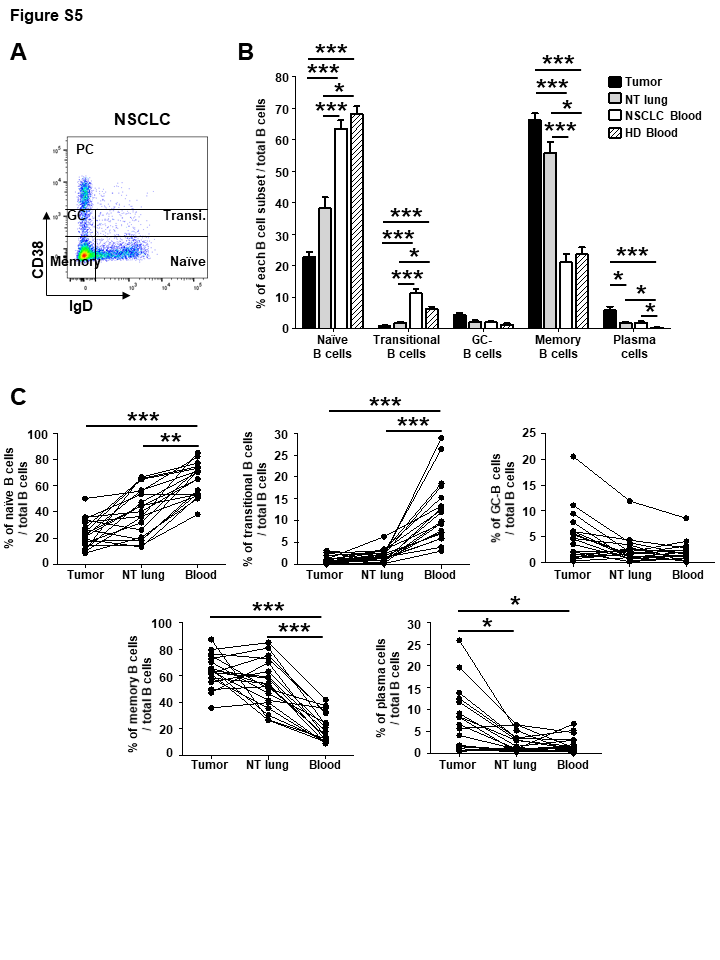

Supplement: Supplementary Figure 5 — Memory B cells and plasma cells are more frequent in tumors than at non-tumor sites. (A) Representative dot plot of B cell subsets among total CD19+ B cells in NSCLC tumor, based on the differential expression of IgD and CD38 (IgD+ CD38+/− naïve B cells; IgD+ CD38+ pre-GC B cells; IgD− CD38+ GC-B cells; IgD− CD38+/− memory B cells; IgD− CD38++ PC). (B) Histogram represents the percentages of each B cell subset among total CD19+ B cells in NSCLC tumor, NT lung, NSCLC peripheral blood and healthy donor peripheral blood (mean ± SEM) sites. P-values were calculated with one-way ANOVA/Kruskal-Wallis/Dunn's test. (C) Graphs represent the percentages of each B cell subset among total B cells, in each compartment of NSCLC patients. P-values were calculated with one-way ANOVA/Friedman/Dunn's test. *P < 0.05, **P < 0.005, ***P < 0.001. GC, germinal center; HD, healthy donor; NSCLC, non-small-cell lung cancer; NT, non-tumor; pre-GC, pre-germinal center; PC, plasma cell. [file Image_5.tif]

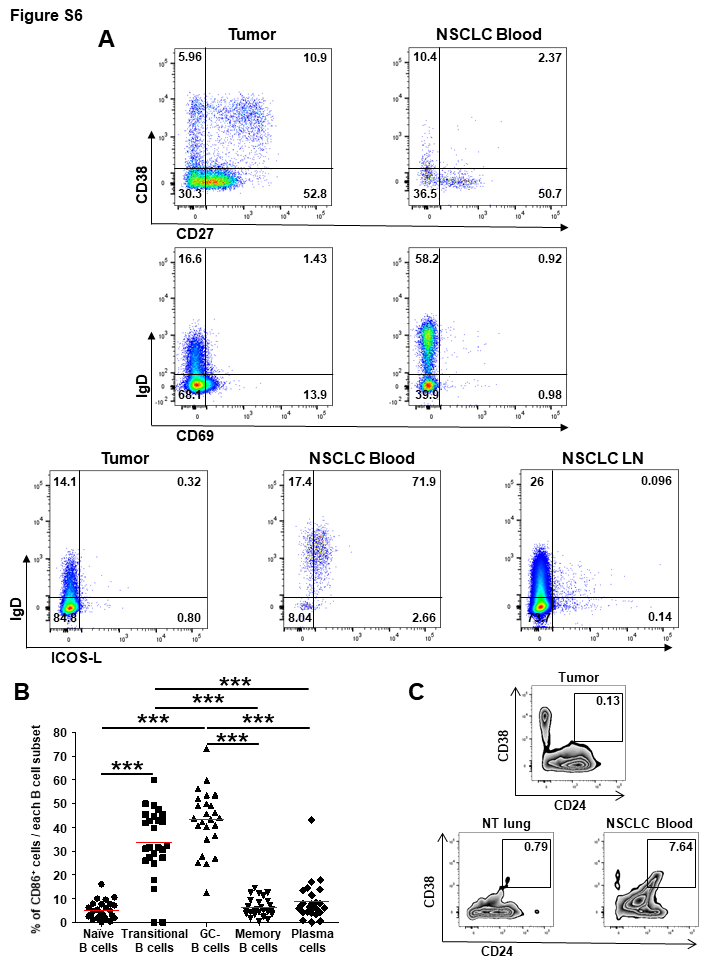

Supplement: Supplementary Figure 6 — Analysis of B cell subsets in different anatomical sites. (A) Representative dot plots of expression of CD27/CD38 (upper panels), CD69/IgD (middle panels), and ICOS-L/IgD (lower panels) among total CD19+ B cells in NSCLC tumors (left panels) or NSCLC peripheral blood and lymph nodes. Percentages of cells are indicated in each quadrant. (B) Frequencies of CD86+ cells among each B cell subset in NSCLC tumors. Means are indicated by horizontal red lines. P-values were calculated with one-way ANOVA/Kruskal-Wallis/Dunn's test. ***p < 0.001. (C) Representative dot plot of regulatory B cells (Bregs) among total CD19+ B cells from NSCLC tumor (upper panel), non-tumor lung (lower left panel) or NSCLC peripheral blood (lower right panel) sites, based on the differential expression of CD38 and CD24. Percentages of CD38high CD24high Bregs are indicated in the corresponding gate. GC, germinal center; LN, lymph node; NSCLC, non-small-cell lung cancer; pre-GC, pre-Germinal Center; NT, non-tumor; PC, plasma cell. [file Image_6.tif]

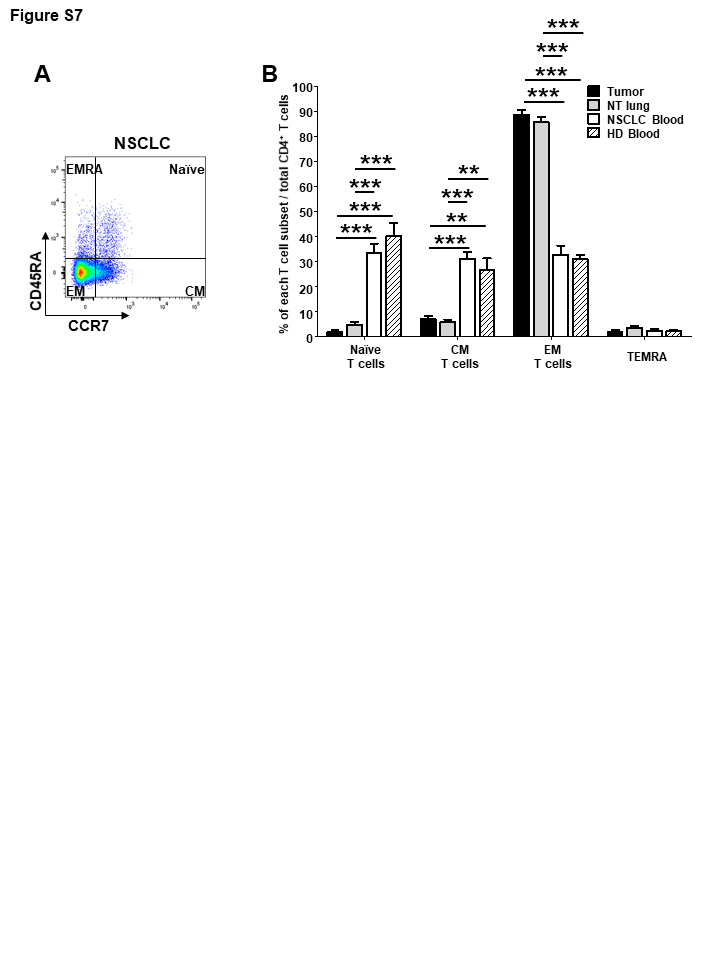

Supplement: Supplementary Figure 7 — Tumors have higher frequencies of effector-memory CD4+ T cells than non-tumor cells. (A) Representative dot plot of T cell subsets among total CD3+ CD4+ T cells in NSCLC tumors, based on the differential expression of CD45RA and CCR7 (CD45RA+ CCR7+ naïve T cells; CD45RA− CCR7+ central-memory (CM) T cells; CD45RA− CCR7− effector-memory (EM) T cells; CD45RA+ CCR7− terminally differentiated CD45RA+ effector cells (TEMRA)). (B) Histogram represents the percentages of each T cell subset among total CD3+ CD4+ T cells from NSCLC tumor, non-tumor lung (NT), NSCLC peripheral blood and healthy donor (HD) peripheral blood (mean ± SEM) sites. P-values were calculated with one-way ANOVA/Kruskal-Wallis/Dunn's test. *p < 0.05, **p < 0.005, ***p < 0.001. CM, central-memory; EM, effector-memory; HD, healthy donor; NSCLC, non-small-cell lung cancer; NT, non-tumor; TEMRA, terminally differentiated CD45RA+ cell. [file Image_7.tif]

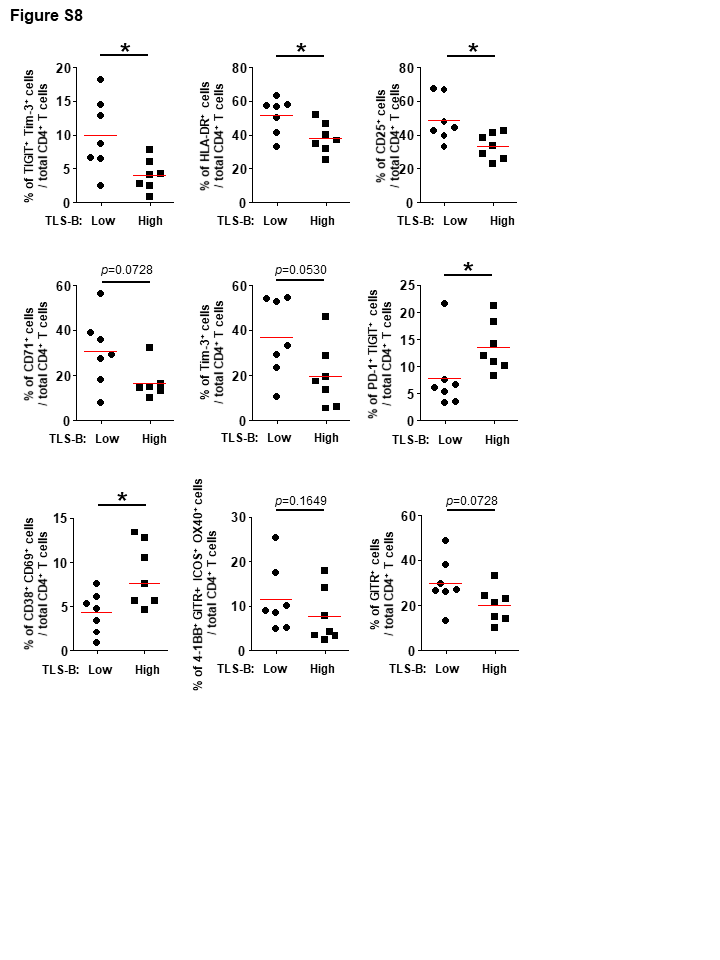

Supplement: Supplementary Figure 8 — Correlation between TLS-B cell density and specific CD4+ T cell markers in tumors. Graphs represent the frequencies of cells expressing the mentioned markers among TIL CD3+ CD4+ T cells with tumors stratified into TLS-BLow (n = 7, first quartile) and TLS-BHigh (n = 7, last quartile) groups. Means are indicated by horizontal red lines. P-values were calculated with the Mann Whitney test. *p < 0.05. ns, non-significant. [file Image_8.tif]

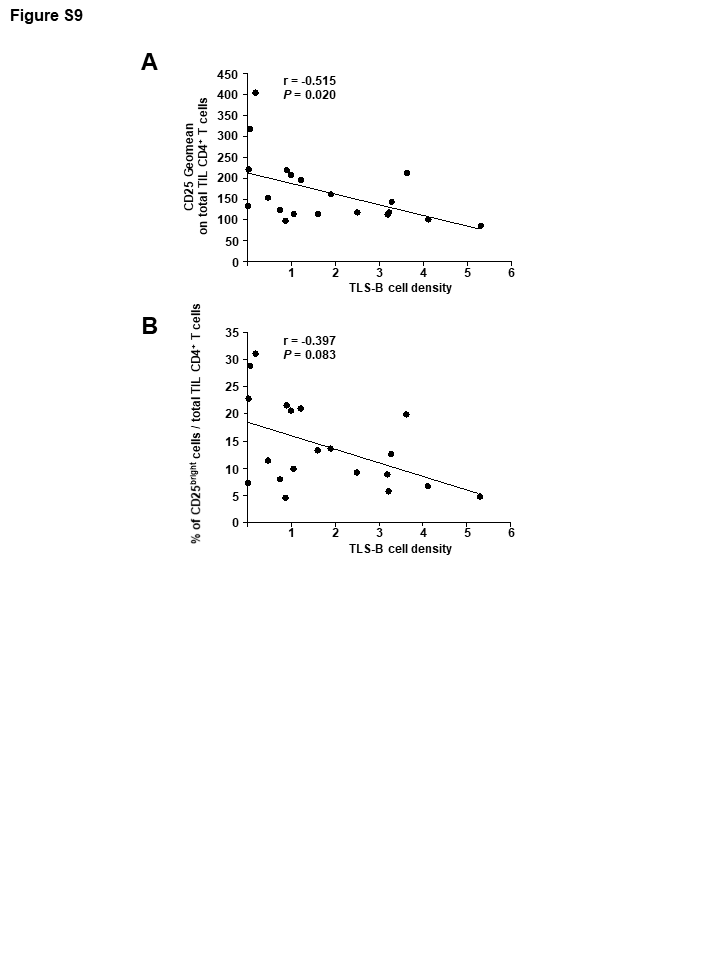

Supplement: Supplementary Figure 9 — Lower percentages of CD25bright cells/total CD4+ T cells in TLS-Bhigh vs. TLS-Blow NSCLC tumors. Correlations between (A) CD25 geomean on intratumor CD3+ CD4+ T cells (n = 20) or (B) the percentage of CD25brigh T cells among intratumor CD3+ CD4+ T cells (n = 20) with TLS-B cell density in the corresponding tumor sections. Statistical significance was determined by the Spearman test. [file Image_9.tif]

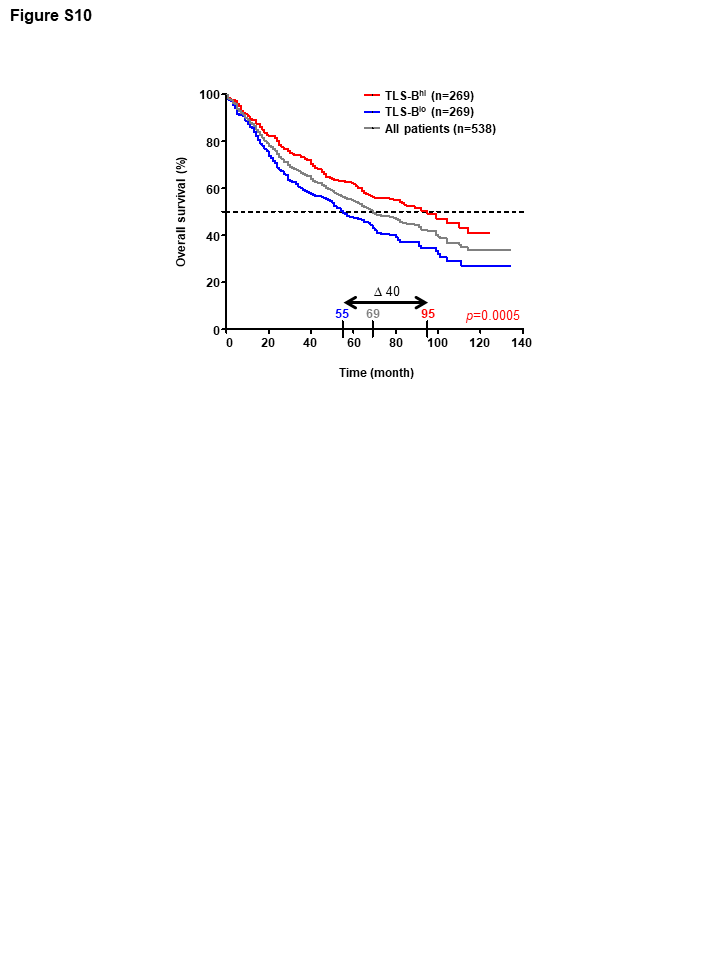

Supplement: Supplementary Figure 10 — High density of TLS-B cells is associated with better overall survival of NSCLC patients. Kaplan-Meier curves of overall survival (OS) among the retrospective cohort (n = 538 patients) according to TLS-B cell density (using median cut-off value). The horizontal dashed line on each graph represents the median survival. Median survival values for each group of patients are also reported on the graph, as well as the difference in months between the best and worst surviving groups (Δ). P-value was determined using the log-rank test. [file Image_10.tif]

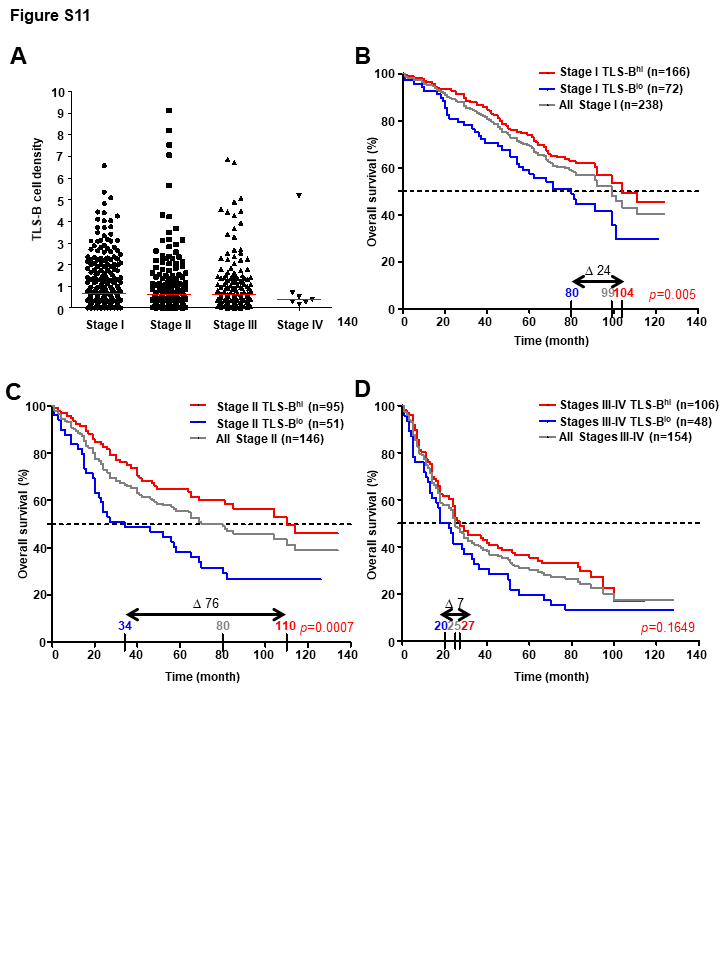

Supplement: Supplementary Figure 11 — No differences in TLS-B cell density between NSCLC tumor stages but prognostic value of TLS-B cell density best in stages I and II. (A) Graph represents the distribution of TLS-B cell densities for different NSCLC stages in the retrospective cohort (n = 538). Median density in each group is indicated by red horizontal lines. Statistical tests used: one-way ANOVA, Kruskal-Wallis test, Dunn's Multiple Comparison. (B–D) Kaplan-Meier overall survival (OS) curves by TLS-B cell density among NSCLC (B) stage I (n = 238), (C) stage II (n = 146), or (D) combined stages III-IV (n = 154). Optimal cut-off values used were determined in the whole retrospective cohort. The horizontal dashed line on each graph represents the median survival. Median survival values for each group of patients are also reported on the graph, as well as the difference in months between the best and worst surviving group (Δ). P-values were determined with the log-rank test. [file Image_11.tif]

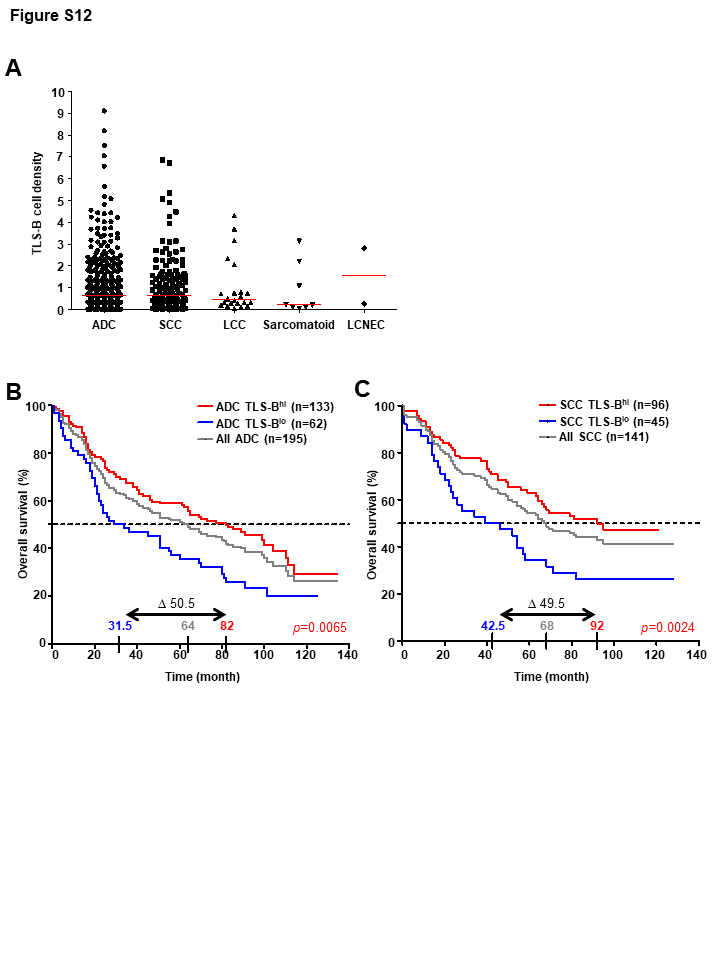

Supplement: Supplementary Figure 12 — Densities and prognostic value of TLS-B cells according to histological subtypes. (A) Graph represents the distribution of TLS-B cell densities among different NSCLC histological subtypes in the retrospective cohort. Median densities in each group are indicated by red horizontal lines. Statistical tests used: one-way ANOVA, Kruskal-Wallis test, Dunn's Multiple Comparison. (B,C) Kaplan-Meier overall survival (OS) curves by TLS-B cell density in patients with (B) adenocarcinoma (ADC, n = 195) or (C) squamous cell carcinoma (SCC, n = 141) NSCLC subtype. The optimal cut-off values used were determined from the entire retrospective cohort (n = 538). The horizontal dashed line on each graph represents the median survival. Median survival values for each group of patients are also reported on the graph, as well as the difference in months between the groups with the best and worst survival (Δ). P-values were determined with the log-rank test. ADC, adenocarcinoma; LCC, large cell carcinoma; LCNEC, large cell neuroendocrine carcinoma; SCC, squamous cell carcinoma. [file Image_12.tif]
