# Supplementary material for: Tertiary Lymphoid Structure-B Cells Narrow Regulatory T Cells Impact in Lung Cancer Patients
Source: Front Immunol. 2021 Mar 8;12:626776. doi: 10.3389/fimmu.2021.626776 (PMC7983944; doi:10.3389/fimmu.2021.626776)
Supplement: Supplementary Table 1 — Clinical and pathological features of 538 NSCLC patients enrolled in the retrospective study. Pathologic staging of lung cancer was determined according to the new TNM staging classification (46). Histological subtypes were determined according to the WHO classification (47). Patients received neither preoperative chemotherapy nor radiotherapy. Patients with mixed histologic features, a T3 tumor, or pleural invasion were ineligible. Others: large cell carcinoma (4%), sarcomatoid carcinoma (1.5%), and large cell neuroendocrine carcinoma (0.5%). ND, not determined. [file Table_1.DOCX]

**Table S1**

| **Characteristics (retrospective cohort)** | **Number (%)** |
| --- | --- |
| **Gender** |  |
| male  female | 419 (78%)  119 (22%) |
| **Age** |  |
| mean (year) ± SEM  range | 63 ± 11  19-84 |
| **Smoking history** |  |
| past/current  never smokers | 476 (88%)  47 (9%) |
| ND | 15 (3%) |
| pack-year ± SEM  range | 42 ± 24  0-150 |
| **Histological subtype** |  |
| Adenocarcinoma | 365 (68%) |
| Squamous cell carcinoma | 141 (26%) |
| Others | 32 (6%) |
| **pTNM stage** |  |
| I | 238 (44%) |
| II | 146 (27%) |
| III+IV | 154 (29%) |
| **Vital status of patients** |  |
| Alive | 226 (42%) |
| Dead | 312 (58%) |

**Table S2**

| **Characteristics (prospective cohort)** | **Number (%)** |
| --- | --- |
| **Gender** |  |
| male  female | 30 (54%)  26 (46%) |
| **Age** |  |
| mean (year) ± SEM  range | 68 ± 8  52-88 |
| **Smoking history** |  |
| past/current  never smoker | 40 (72%)  8 (14%) |
| ND | 8 (14%) |
| pack-year ± SEM  range | 34 ± 22  0-80 |
| **Histological subtype** |  |
| Adenocarcinoma | 27 (48%) |
| Squamous cell carcinoma | 27 (48%) |
| Others | 2 (4%) |
| **pTNM stage** |  |
| I | 20 (36%) |
| II | 18 (32%) |
| III | 18 (32%) |

**Table S3**

| **IHC** | **Conjugate** | **Host** | **Clone or reference** | **Source** | **Antigen retrieval** |
| --- | --- | --- | --- | --- | --- |
| Antibodies: |  |  |  |  |  |
| APAAP | AP | Mouse IgG1 | D0651 | Dako-Agilent | NA |
| CD20 | UC | Mouse IgG2a | L26 | Dako-Agilent | pH 6.0 |
| CD21 | UC | Mouse IgG1 | 1F8 | Dako-Agilent | pH 6.0 |
| CD3 | UC | Rabbit IgG | A0452 | Dako-Agilent | TRS |
| FoxP3 | UC | Mouse IgG1 | 236A/E7 | Abcam | TRS |
| Mouse IgG | AP | Goat F(ab')_2_ | 115-056-062 | Jackson ImmunoResearch | NA |
| Mouse IgG1 | UC | Sheep Ig | AU273 | Binding Site | NA |
| Mouse IgG2a | Biotin | Goat IgG | 115-065-206 | Jackson ImmunoResearch | NA |
| Pan-cytokeratins | UC | Mouse IgG1 | AE1-AE3 | Dako-Agilent | pH 8.0 |
| Reagents: |  |  |  |  |  |
| AEC | NA | NA | SK-4200 | Vector Laboratories | NA |
| DAPI | NA | NA | D3571 | Life Technologies | NA |
| Hematoxylin | NA | NA | 1.09249.2500 | Merck | NA |
| SAP | NA | NA | SK-5300 | Vector | NA |
| Streptavidin | HRP | NA | P039701 | Dako-Agilent | NA |

**Table S4**

| **Flow cytometry** | **Conjugate** | **Host** | **Clone or reference** | **Source** |
| --- | --- | --- | --- | --- |
| Antibodies: |  |  |  |  |
| 4-1BB | BV421 | Mouse IgG1 | 4B4-1 | BioLegend |
| 4-1BBL | PE | Mouse IgG1 | C65-485 | BD Biosciences |
| BTLA | PE | Mouse IgG1 | J168-540 | BD Biosciences |
| CCR7 | PE-Cy7 | Mouse IgG2a | G043H7 | BioLegend |
| CD3 | AF700 | Mouse IgG1 | UCHT1 | BD Biosciences |
| CD3 | PerCP | Mouse IgG1 | UCHT1 | BioLegend |
| CD4 | AF700 | Mouse IgG1 | RPA-T4 | BD Biosciences |
| CD4 | BV605 | Mouse IgG2b | OKT4 | BioLegend |
| CD8 | APC-H7 | Mouse IgG1 | SK1 | BD Biosciences |
| CD8 | BV650 | Mouse IgG1 | RPA-T8 | BioLegend |
| CD19 | ECD | Mouse IgG1 | J3.119 | Beckman Coulter |
| CD24 | BV650 | Mouse IgG2a | ML5 | BD Biosciences |
| CD25 | BV421 | Mouse IgG1 | M-A251 | BD Biosciences |
| CD27 | BV650 | Mouse IgG1 | O323 | BioLegend |
| CD38 | PerCP-eF710 | Mouse IgG1 | HB7 | eBioscience |
| CD40 | Pe-Cy7 | Mouse IgG1 | 5C3 | BioLegend |
| CD40-L | APC-Cy7 | Mouse IgG1 | 24-31 | BioLegend |
| CD45 | PE | Mouse IgG1 | J.33 | Beckman Coulter |
| CD45RA | ECD | Mouse IgG1 | 2H4 | Beckman Coulter |
| CD69 | AF700 | Mouse IgG1 | FN50 | BD Biosciences |
| CD69 | PE | Mouse IgG1 | FN50 | BD Biosciences |
| CD70 | FITC | Mouse IgG3 | Ki-24 | BD Biosciences |
| CD71 | FITC | Mouse IgG2a | M-A712 | BD Biosciences |
| CD80 | BV605 | Mouse IgG1 | 2D10 | BioLegend |
| CD83 | PE.Cy7 | Mouse IgG1 | HB15e | BD Biosciences |
| CD86 | AF700 | Mouse IgG1 | 2331 | BD Biosciences |
| CD95 | APC | Mouse IgG1 | DX2 | BD Biosciences |
| CD227 | FITC | Mouse IgG1 | HMPV | BD Biosciences |
| CTLA-4 | APC | Mouse IgG1 | L3D10 | BioLegend |
| Epithelial antigen | FITC | Mouse IgG1 | Ber-EP4 | Dako-Agilent |
| FoxP3 | AF700 | Rat IgG2a | PCH101 | eBioscience |
| GITR | AF488 | Mouse IgG1 | AITR | eBioscience |
| GITR-L | APC | Mouse IgG1 | 109101 | R&D |
| HLA-DR | APC-Cy7 | Mouse IgG2a | L243 | BioLegend |
| ICOS | PerCP-eF710 | Mouse IgG1 | ISA3 | eBioscience |
| ICOS-L | FITC | Mouse IgG1 | MIH11 | Clinisciences |
| IgD | BV421 | Mouse IgG2a | IA6-2 | BD Biosciences |
| LAG-3 | FITC | Mouse IgG1 | 17B4 | EnzoLifesciences |
| OX40 | PE | Mouse IgG1 | ACT35 | BD Biosciences |
| OX40-L | PE | Mouse IgG1 | 11c3.1 | BioLegend |
| Pan-cytokeratins | AF488 | Mouse IgG1 | AE1-AE3 | eBioscience |
| PD-1 | APC-Cy7 | Mouse IgG1 | EH12.2H7 | BioLegend |
| Tim-3 | BV421 | Mouse IgG1 | F38-2E2 | BioLegend |
| TIGIT | PerCP-eF710 | Mouse IgG1 | MBSA43 | eBioscience |
| Reagent: |  |  |  |  |
| Viability marker | NA | NA | L34959 | Invitrogen |
